# Targeting the miRNA-155/TNFSF10 network restrains inflammatory response in the retina in a mouse model of Alzheimer’s disease

**DOI:** 10.1038/s41419-021-04165-x

**Published:** 2021-10-05

**Authors:** Chiara Burgaletto, Chiara Bianca Maria Platania, Giulia Di Benedetto, Antonio Munafò, Giovanni Giurdanella, Concetta Federico, Rosario Caltabiano, Salvatore Saccone, Federica Conti, Renato Bernardini, Claudio Bucolo, Giuseppina Cantarella

**Affiliations:** 1 Department of Biomedical and Biotechnological Sciences, Section of Pharmacology, University of Catania School of Medicine, Catania, Italy; 2 Department of Biomedical and Biotechnological Sciences, Section of Biochemistry, University of Catania School of Medicine, Catania, Italy; 3 Department of Biological, Geological and Environmental Sciences, Section of Animal Biology, University of Catania, Catania, Italy; 4 Department Gian Filippo Ingrassia, Section of Anatomic Pathology, University of Catania, Catania, Italy; 5 Clinical Toxicology Unit, University Hospital, University of Catania, Catania, Italy

**Keywords:** Alzheimer's disease, Neurodegeneration

## Abstract

Age-related disorders, such as Alzheimer’s disease (AD) and age-related macular degeneration (AMD) share common features such as amyloid-β (Aβ) protein accumulation. Retinal deposition of Aβ aggregates in AMD patients has suggested a potential link between AMD and AD. In the present study, we analyzed the expression pattern of a focused set of miRNAs, previously found to be involved in both AD and AMD, in the retina of a triple transgenic mouse model of AD (3xTg-AD) at different time-points. Several miRNAs were differentially expressed in the retina of 3xTg-AD mice, compared to the retina of age-matched wild-type (WT) mice. In particular, bioinformatic analysis revealed that miR-155 had a central role in miRNA-gene network stability, regulating several pathways, including apoptotic and inflammatory signaling pathways modulated by TNF-related apoptosis-inducing ligand (TNFSF10). We showed that chronic treatment of 3xTg-AD mice with an anti-TNFSF10 monoclonal antibody was able to inhibit the retinal expression of miR-155, which inversely correlated with the expression of its molecular target SOCS-1. Moreover, the fine-tuned mechanism related to TNFSF10 immunoneutralization was tightly linked to modulation of TNFSF10 itself and its death receptor TNFRSF10B, along with cytokine production by microglia, reactive gliosis, and specific AD-related neuropathological hallmarks (i.e., Aβ deposition and Tau phosphorylation) in the retina of 3xTg-AD mice. In conclusion, immunoneutralization of TNFSF10 significantly preserved the retinal tissue in 3xTg-AD mice, suggesting its potential therapeutic application in retinal degenerative disorders.

## Introduction

Alzheimer’s Disease (AD) is an age-related neurodegenerative disorder, whose onset precedes the disease’s symptoms and diagnosis. Since it was first described, big efforts have been made to validate approaches for early diagnosis, along with effective treatments for AD [1].

Despite the advent of sophisticated neuroimaging techniques and the search for reliable biomarkers, to date, the definitive diagnosis of AD can only be made after the post-mortem identification of amyloid-β (Aβ) plaques and neurofibrillary tangles in the brain of AD patients [2].

Research studies also focused on AD diagnosis through ophthalmic diagnostic procedures because the eye is considered an easily reachable “window to the brain”. Furthermore, the retina, a central nervous system tissue formed as a developmental outgrowth of the brain, is profoundly affected by AD [3].

Aβ deposition is considered a hallmark of AD pathology, and retinal Aβ deposits reported in AD patients and early-stage cases matched with brain amyloid pathology [4–6].

Furthermore, visual deficits and retinal ultrastructural modifications, such as ganglion cell degeneration, nerve fiber layer (NFL) thinning and optic nerve degeneration, were experienced by AD patients [6–8], strengthen the hypothesis that the retina represents a valuable site of pre-symptomatic AD stage imaging, and at the preclinical level can be considered as a surrogate tissue to be analyzed for mechanistic studies [9].

Noteworthy, studies on several retinal degenerative diseases such as glaucoma and age-related macular degeneration (AMD), which share some features with AD [10–13], may provide some clues to understand the pathological process underlying such disorder.

Despite major advances in understanding mechanisms of AD, to date, there are no disease-modifying options available to slow down or halt the progression of the neurodegenerative process. Current pharmacological treatments only transiently mitigate the severity of symptoms, with generally unsatisfactory clinical outcomes [14].

In such scenario, it would be helpful to identify reliable targets for AD therapeutic intervention [15].

Besides a leading role as feasible disease biomarkers, microRNAs (miRNAs) expression profiles could provide an overview of the complex network of molecular pathways in AD and AMD, which is defined as the “dementia of the eye” [12].

In this light, our research group has previously identified an overlap in the expression patterns of specific miRNAs (miR-155, miR-126a, miR-23a, miR-34a, miR-9, miR-27a, miR-146a), between the retina of a rat model of AMD (Aβ intravitreal injection) and serum of AMD patients, which were also recognized as potentially useful biomarkers of AD pathology [11, 16].

It is well known that chronic neuroinflammation is one of the prominent hypotheses put forward to describe the pathogenesis of AD [17]. Several miRNA networks, including miRNAs related with innate immunity and neuroinflammation, have been found to be dysregulated in AD [18, 19].

In fact, as previously reported, miR-155 upregulation contributes to neuroinflammation in AD [20], where it plays a central role in the regulation of the innate immune response through the modulation of cytokines and chemokines production [21, 22]. In this regard, cytokine Tumor necrosis factor-related apoptosis-inducing ligand (TRAIL), a cytokine formerly known as TNFSF10 and a member of the TNF superfamily, produced by injured neurons [23] and by activated glia [24], with its potent immune-modulatory properties represents a pleiotropic fine-tuning effector of the inflammatory/immune response with an orchestrating role in the complex scenario of AD etiopathogenesis [25]. TNFSF10 mediates death signaling through interaction with its death receptors TNFRSF10B (DR5) and TNFRSF10A (DR4), and interferes with several pathways, including the Wnt pathway concurring to neuronal damage [26].

The prominent role of TNFSF10 in Aβ-related neurodegeneration has been already demonstrated in different studies showing that neutralization of TNFSF10 pathway in an in vitro model of AD protects human neuronal cell line from Aβ−neurotoxicity [23]; as well as in the triple transgenic mouse model of AD (3xTg-AD) where it exerts a beneficial effect on central and peripheral AD-related inflammatory/immune response and disease outcome [27, 28].

Given the emerging role of miRNAs and TNFSF10 system in AD-associated neuroinflammation and considering that the retina is an integral part of the central nervous system originating from the neural tube, in the present study, we investigated the expression of a focused set of miRNAs, linked to AD and AMD, in the retina of 3xTg-AD mice. Furthermore, we investigated the combinatorial effect of miRNAs through bioinformatic approaches. We highlighted a direct link between these miRNAs and the TNFSF10 signaling pathway in AD-related inflammation, and to support our experimental hypothesis we used the retina as a surrogate tissue for mechanistic and pharmacological studies in AD pathology. Furthermore, our pre-clinical data evidenced that anti-TNFSF10 antibody treatment would be of value for the management of sight-threatening retinal degenerative diseases.

## Results

### 3xTg-AD retinal miRNA expression and bioinformatic analysis of related biochemical pathways

In light of the reported link between AD and retinal degeneration associated with AMD [10] and to identify useful biomarkers of disease progression, we analyzed the expression of a specific set of miRNAs, involved in both disease conditions, in retina extracts from 3xTg-AD mice at different time-points (3, 9 and 15 months of age), resembling the evolution of AD-like pathology.

This focused set of miRNAs was chosen with the rationale that it was altered in sera of AMD patients as well as in the retina of rats subjected to intravitreal injection of Aβ oligomers [11], thus resembling a model of early AMD.

Real-time PCR analyses highlighted five miRNAs (miR-155, miR-126a, miR-23a, miR-34a, miR-27a) significantly dysregulated in the retina of 3xTg-AD mice compared to age-matched wild-type (WT) mice, while the miR-9 expression levels were not significantly modulated.

**Fig. 1:**
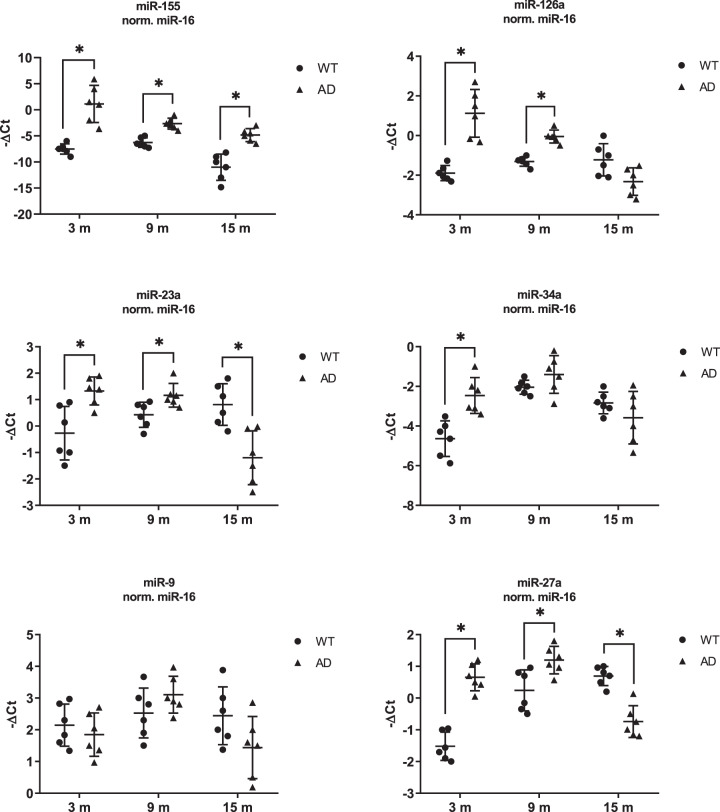
Differential expression analysis of miRNAs in the retina of 3xTg-AD mice. RT-qPCR was performed to determine the expression of miR-155, miR-126a, miR-23a, miR-34a, miR-9, miR-27a in the retinas from 3xTg-AD mice at three different (3-, 9-, and 15-month-old) age periods. Data are expressed as mean ± standard deviation. One-way ANOVA and Tukey’s multiple comparisons test were used to determine statistical significance. *p < 0.05 *vs*. WT age-matched mice. *N* = 6 animals; 6 independent retinal samples, 2 pooled retinas per sample in each group.

Noteworthy, we found that miR-155 was significantly up-regulated in the retina of the AD mice at all ages (Fig. 1). Retinal miR-126a was significantly up-regulated in the retina of 3- and 9-month-old 3xTg-AD mice, while miR-126a expression level decreased, though not significantly, in 15-month-old 3xTg-AD mice compared to age-matched WT mice. A similar trend was observed for miR-23a and miR-27a expression levels, that after upregulation, significantly decreased in the retinas of 15-month-old 3xTg-AD compared to WT mice. The miR-34a retinal level was significantly up-regulated only in 3-month-old AD mice, compared to WT.

**Fig. 2:**
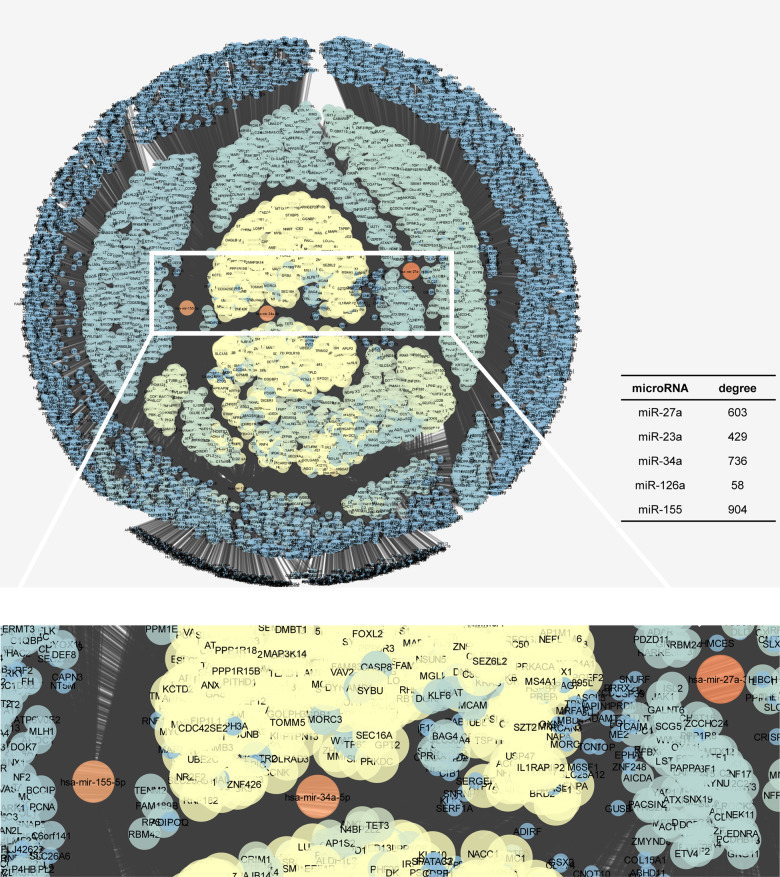
The miRNA-gene network predicted by miRNet analysis. The predicted network (Prefuse force directed layout based on edge betweenness) included about 20000 edges (connections) and 10000 nodes (miRNAs or genes). Only three nodes showed the highest degree (included table) and particularly the highest betweenness centrality (red color). From left to right, the red nodes represent miR-155, miR-34a, and miR-27a, bearing also the highest degree values. These mentioned node parameters strictly influence the stability of the network. Centrality metric analyses were carried out with Cytoscape and network parameters were plotted in the graphic representation: closeness centrality (proportional to node dimension), betweenness centrality (temperature color scale, blue < red), edge betweenness (proportional to edge thickness).

To shed light on the biological effects of miRNAs expression patterns, several bioinformatic approaches were carried out. We hereby predicted the pathways dysregulated by the analyzed miRNAs accessing the miRNet webserver, which generated a complex network of about 17,000 interactions (edge). Degree centrality analysis with Cytoscape has shown that miR-155 is the node with the highest node degree value (Fig. 2), demonstrating that within about 2,000 nodes, the miR-155 displays the highest number of incident links with other nodes, which represent target genes. Moreover, miR-155 has shown the highest betweenness centrality, along with miR-34a and miR-27a than other miRNAs.

To carry out a straightforward analysis of this complex miRNA-gene interaction network, we have done a KEGG (Kyoto Encyclopedia of Genes and Genomes) enrichment analysis (Supplementary Table 1) [29]. Top predicted pathways were: “Apoptosis”, “T cell receptor signaling pathway”, “p53 signaling pathway”, “Neurotrophin signaling pathway”, “Alzheimer’s disease”, “Natural killer cell-mediated cytotoxicity”, “Cytokine-cytokine receptor interaction”. Interestingly, the miRNet enriched analysis, for the given miRNA-gene network, has yielded two diseases: “Inflammation” and “Alzheimer’s Disease”.

Computational analysis of the combinatorial effects among this group of miRNAs, specifically miR-155-5p, miR-126-3p and miR-23a-3p revealed that these miRNAs target also genes belonging to the TNF-related apoptosis-inducing ligand (TNFSF10)-mediated apoptotic signaling pathway (Supplementary Fig. 1), including the TNFSF10 death receptors TNFRSF10B and TNFRSF10A. Additionally, dysregulated miRNAs were predicted to modulate other pathways through targeting TNFSF10 related genes: “p53 signaling pathway”, “Cytokine-Cytokine receptor interaction” and “Natural killer cell-mediated cytotoxicity”. In Table 1 we evidenced the combinatorial effect of miRNAs on TNFSF10 related genes. Specifically, Table 1 shows experimentally validated miRNA:mRNA interactions, as regards as Tarbase algorithm output. Noteworthy, both human TNFRSF10B and TNFRSF10A are experimentally validated targets of miR-155. Looking at the time-dependent pattern of expression of miRNAs obtained in 3xTg-AD mice, we observed that in young mice (3- and 9-month-old mice), most of the miRNAs up-regulated in the retina of AD mice could negatively regulate the expression of the TNFSF10 pathway target genes. On the contrary, in the late phase (15-month-old mice), significantly downregulated miRNAs such as miR-23a and miR-27a could act as positive regulators of the TNFRSF10B receptor and FADD, likely promoting their detrimental effects on the retina.

**Table 1:** miRNAs-TNFSF10 signaling pathways interactions in retina of 3xTg-AD mice.

	3-month-old 3xTg-AD mice	9-months-old 3xTg-AD mice	15-month-old 3xTg-AD mice
**miR-155-5p** ***TNFRSF10B*** ***TNFRSF10A***	↑* miRNA fold regulation ↓ predicted target regulation	↑* miRNA fold regulation ↓ predicted target regulation	↑* miRNA fold regulation ↓ predicted target regulation
**miR-126-3p** ***TNFRSF10B***	↑* miRNA fold regulation ↓ predicted target regulation	↑* miRNA fold regulation ↓ predicted target regulation	↓ miRNA fold regulation ↑ **predicted target regulation**
**miR-23a-3p** ***TNFRSF10B***	↑* miRNA fold regulation ↓ predicted target regulation	↑ * miRNA fold regulation ↓ predicted target regulation	↓* miRNA fold regulation ↑ **predicted target regulation**
**miR-34a-5p** ***TNFRSF10D***	↑* miRNA fold regulation ↓ predicted target regulation	↑ miRNA fold regulation ↓ predicted target regulation	↓ miRNA fold regulation ↑ **predicted target regulation**
**miR-27a-3p** **FADD**	↑* miRNA fold regulation ↓ predicted target regulation	↑* miRNA fold regulation ↓ predicted target regulation	↓* miRNA fold regulation ↑ **predicted target regulation**

Note: * *p* < 0.05 *vs*. WT age-matched mice. Bold characters highlight that most of dysregulated miRNAs in the retina of 15-month-old 3xTg-AD mice, are down-regulated, leading to putative up-regulation of experimentally validated targets of TNFSF10 pathway. In humans, TNFRSF10A is a target of miR-155.

### Neutralization of TNFSF10 modulates the expression of miR-155 and SOCS-1 in the retinas of 3xTg-AD mice

**Fig. 3:**
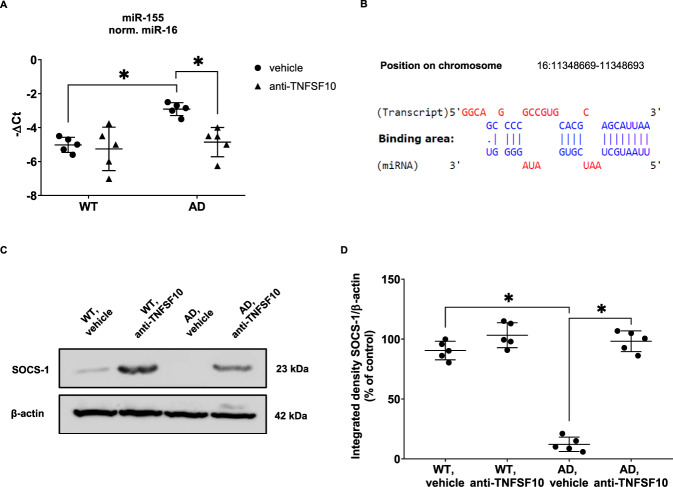
Anti-TNFSF10 treatment decreased miR-155 retinal levels in 15-month-old 3xTg-AD mice. **A** RT-qPCR was performed to determine the retinal expression of miR-155-5p in 15-month-old 3xTg-AD mice treated with anti-TNFSF10. One-way ANOVA and post-hoc Tukey’s multiple comparisons test were used. *N* = 5 animals; 5 independent retinal samples, 2 pooled retinas per sample in each group. **B** Bioinformatic prediction of SOCS-1 mRNA binding with miR-155-5p. **C** Western blot analysis was performed to evaluate the expression of the miR-155-5p molecular target SOCS-1 in the retinas of 3xTg-AD mice. **D** Densitometric analysis of western blots. Data are expressed as mean ± standard deviation. One-way ANOVA and post-hoc Tukey’s multiple comparisons test were used to determine statistical significance. *p < 0.05. *N* = 5 animals; 5 independent retinal samples, 2 pooled retinas per sample in each group.

Bioinformatic analysis relied on already validated in-vitro functional assays and correlates dysregulated miRNAs in AD retina with the TNFSF10-signaling pathway. To confirm bioinformatic data in in-vivo studies, we focused our analysis on 15-month-old 3xTg-AD mice which exhibit most of the neuropathological features of the disease. Interestingly, we observed that chronic treatment with an anti-TNFSF10 monoclonal antibody significantly inhibited only the expression of miR-155 (Fig. 3A). No significant effect on miR-155 expression levels was detectable in the retina of WT mice. Other miRNAs from the analyzed set were not significantly differentially expressed when comparing treated with untreated animals.

It has been reported that the suppressor of cytokine signaling 1 (SOCS-1) is a validated (Tarbase v8 algorithm) [30] and predicted target (microTG algorithm) [31] of miRNA-155 (Fig. 3B). Therefore, given that anti-TNFSF10 treatment modulated miR-155 (Fig. 3A), we also investigated the effect of the TNFSF10 immunoneutralization on the expression of its molecular target SOCS-1 in the retinas of the same AD animals. Consistently, western blot analysis revealed that the increased expression of miR-155 in the retina of 15-month-old 3xTg-AD mice was paralleled by a significant decrease of SOCS-1 expression, whereas treatment with anti-TNFSF10 antibody restored SOCS-1 to basal levels (Fig. 3C, D). These data provided the in-vivo functional validation of the tight link between miR-155 and TNFSF10 signaling pathway, along with the *in-silico* analysis carried out on the basis of experimental validated miRNA:mRNA interactions.

### Histological evidence of the efficacy of the anti-TNFSF10 treatment upon the retinal tissue alteration in 3xTg-AD mice

With the aim to verify the role of TNFSF10 immunoneutralization on morphological changes in the retinas of AD mice and to confirm bioinformatic predictions and biomolecular findings, hematoxylin-eosin staining was performed upon retinal sections of 3xTg-AD and WT mice.

**Fig. 4:**
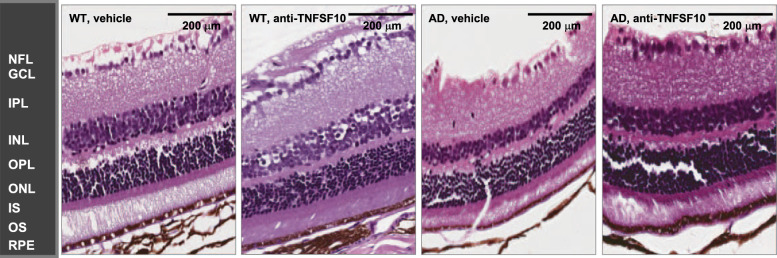
TNFSF10-neutralizing antibody treatment preserved retinal structure in 15-month-old 3xTg-AD mice. Hematoxylin and eosin staining of retinal tissue of WT and 3xTg-AD mice were performed to analyze retina morphological changes following chronic treatment with vehicle or TNFSF10-neutralizing antibody. Original magnification, x200. Scale bar = 200 µm. *N* = 5 animals; 5 independent retinal samples per group. *NFL* nerve fiber layer, *GCL* ganglion cell layer, *IPL* inner plexiform layer, *INL* inner nuclear layer, *OPL* outer plexiform layer, *ONL* outer nuclear layer, *IS* inner segment; *OS* outer segment, *RPE* retinal pigment epithelial.

While no significant changes throughout the retinal layers were observed in specimens from both treated or untreated WT animals, on the other hand, vacuolization and cell disorganization, as well decreased tissue cellularity were observed in the retinal ganglion cell layer (GCL), along with a reduced thickness of the NFL in untreated 3xTg-AD mice. Both tissue parameters appeared improved in the retinas of 3xTg-AD mice treated for twelve months with anti-TNFSF10 treatment, suggesting its neuroprotective effect (Fig. 4).

### TNFSF10 immunoneutralization brings about downregulation of expression of TNFSF10 and its receptor TNFRSF10B in the retina of 3xTg-AD mice

Since it is known that TNFSF10 and its death receptor TNFRSF10B were specifically upregulated in the brain of 3xTg-AD mice [27], and given that the retina is regarded as a developmental outgrowth of the brain, we explored the role of both mediators in the retinas of 3xTg-AD mice treated chronically with an anti-TNFSF10 antibody.

**Fig. 5:**
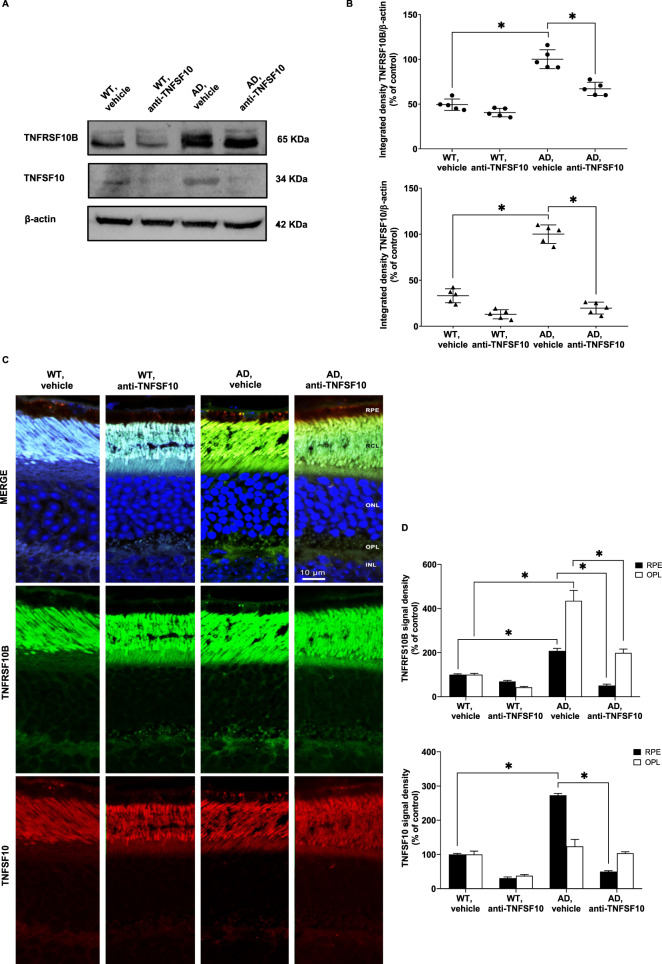
Anti-TNFSF10 treatment modulated retinal expression of TNFSF10 and its TNFRSF10B receptor in 3xTg-AD mice. **A** Immunoblots of retinal lysates for the expression of TNFRSF10B and TNFSF10 proteins. **B** Densitometric analysis of western blots. Data are expressed as mean ± standard deviation. One-way ANOVA and post-hoc Tukey’s multiple comparisons test were used for statistical analysis. *p < 0.05. *N* = 5 animals; 5 independent retinal samples, 2 pooled retinas per sample in each group. **C** Immunohistochemical staining for TNFSF10 and its receptor TNFRSF10B in the retina of WT and 3xTg-AD mice, treated either with vehicle or anti-TNFSF10 antibody. Original magnification, x63. Scale bar = 10 µm. **D** Densitometric analysis of the TNFRSF10B and TNFSF10 immunofluorescence signal in the RPE and OPL retinal layers. Data are expressed as mean ± standard deviation. One-way ANOVA and post-hoc Tukey’s multiple comparisons test were used for statistical analysis. *p < 0.05. *N* = 5 animals; 5 independent retinal samples per group. For each retinal section, 14 optical fields were analyzed.

Western blot analysis revealed that while both TNFSF10 and its death receptor TNFRSF10B were highly expressed in the retinas of untreated 3xTg-AD mice, their expression was significantly attenuated following treatment with an anti-TNFSF10 antibody (Fig. 5A, B).

Biochemical data were confirmed by confocal microscopy experiments, showing that both TNFSF10 and its death receptor TNFRSF10B were highly represented throughout the retina of 3xTg-AD mice, and particularly in the retinal pigmented epithelium (RPE) and the outer plexiform (OPL) layers. While the expression of TNFRSF10B receptor was significantly reduced in the retinal RPE and OPL layers the expression of TNFSF10 was significantly blunted only in the retinal RPE layer of anti-TNFSF10 treated 3xTg-AD mice (Fig. 5C, D, Supplementary Fig. 2). These proteins colocalized in both the retinal RPE and OPL layers of 3xTg-AD mice (Supplementary Fig. 3).

### Inhibition of the TNFSF10 signaling pathway protects the retina of 3xTg-AD mice from neuroinflammatory damage

Neurodegeneration-related breaking of the balance between neurotoxic and neuroprotective mechanisms can induce activation of microglia, which can polarize assuming a classical proinflammatory phenotype, or the alternative anti-inflammatory phenotype via cytokine production [32].

A skewed M1 activation over M2 markedly promotes both AD progression and retinal degeneration, and modulation of microglia polarization has been regarded to as a potential therapeutic target for neuroprotection [25, 33, 34].

**Fig. 6:**
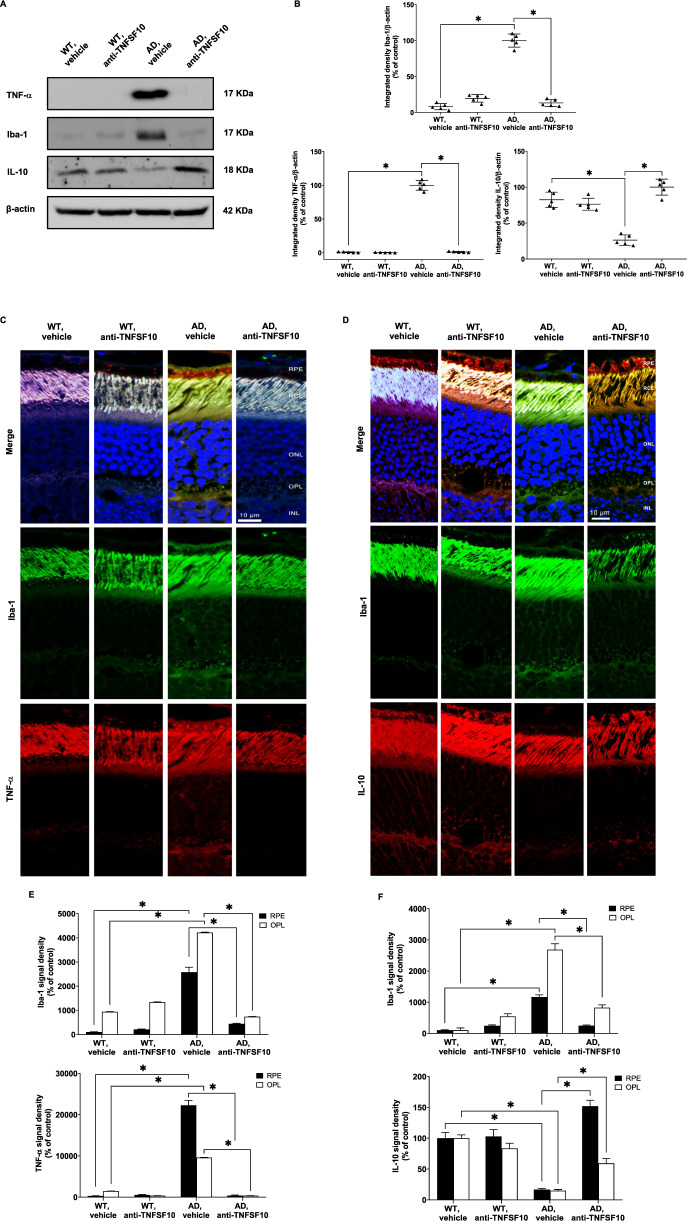
Anti-TNFSF10 treatment inhibited pro-inflammatory microglia activation in the outer-plexiform and in the RPE layers of 3xTg-AD mouse retina. **A** Western blots for TNF-α, Ιba-1 and IL-10 protein expression in the retinas of 3xTg-AD mice, following chronic treatment with an anti-TNFSF10 monoclonal antibody or vehicle. **B** Densitometric analysis of western blots. Data are expressed as mean ± standard deviation. One-way ANOVA and post-hoc Tukey’s multiple comparisons test were used for statistical analysis. *p < 0.05. *N* = 5 animals; 5 independent retinal samples, 2 pooled retinas per sample in each group. **C** Immunohistochemical staining for TNF-α, Iba-1 in the retina of 3xTg-AD mice, treated with either vehicle or anti-TNFSF10 antibody. Original magnification, x63. Scale bar = 10 µm. **D** Immunohistochemical staining for Iba-1, IL-10 in the retina of WT and 3xTg-AD mice, treated with either vehicle or anti-TNFSF10 antibody. Original magnification, x63. Scale bar = 10 µm. **E** Densitometric analysis of the Iba-1, and TNF-α immunofluorescence signal in the RPE and OPL retinal layers. **F** Densitometric analysis of the Iba-1, and IL-10 immunofluorescence in the RPE and OPL retinal layers. Data are expressed as mean ± standard deviation. One-way ANOVA and post-hoc Tukey’s multiple comparisons test were used for statistical analysis. *p < 0.05. *N* = 5 animals; 5 independent retinal samples per group. For each retinal section, 14 optical fields were analyzed.

Concerning the activation status of microglia and related proinflammatory molecules, western blot analysis revealed that, while the expression of microglial marker Iba-1 and of TNF-α was substantially present in the retina of 3xTg-AD mice, anti-TNFSF10 treatment significantly blunted their expression (Fig. 6A, B). Consistently, confocal microscopy analysis showed an increased expression of both TNF-α and Iba-1 (Fig. 6C, E), which, colocalized in both the RPE and OPL layers (Supplementary Fig. 4) of untreated 3xTg-AD mice. Treatment with anti-TNFSF10 restored TNF-α and Iba-1 to basal levels (Fig. 6C, E, Supplementary Fig. 5A).

On the other hand, although Iba-1 is expressed in all retinal layers of both treated and untreated AD mice, the anti-inflammatory cytokine IL-10 is strongly expressed and colocalized with Iba-1 in the RPE and OPL retinal layers of mice treated with anti-TNFSF10 (Fig. 6D, F, Supplementary Fig. 5B, Supplementary Fig. 6). Therefore, anti-TNFSF10 treatment promoted an anti-inflammatory phenotype in microglial cells confirming the western blot data (Fig. 6A, B). These data suggest that TNFSF10 neutralization boosts anti-inflammatory microglia as a consequence of inflammatory microglia inhibition, resulting in retinal protection.

**Fig. 7:**
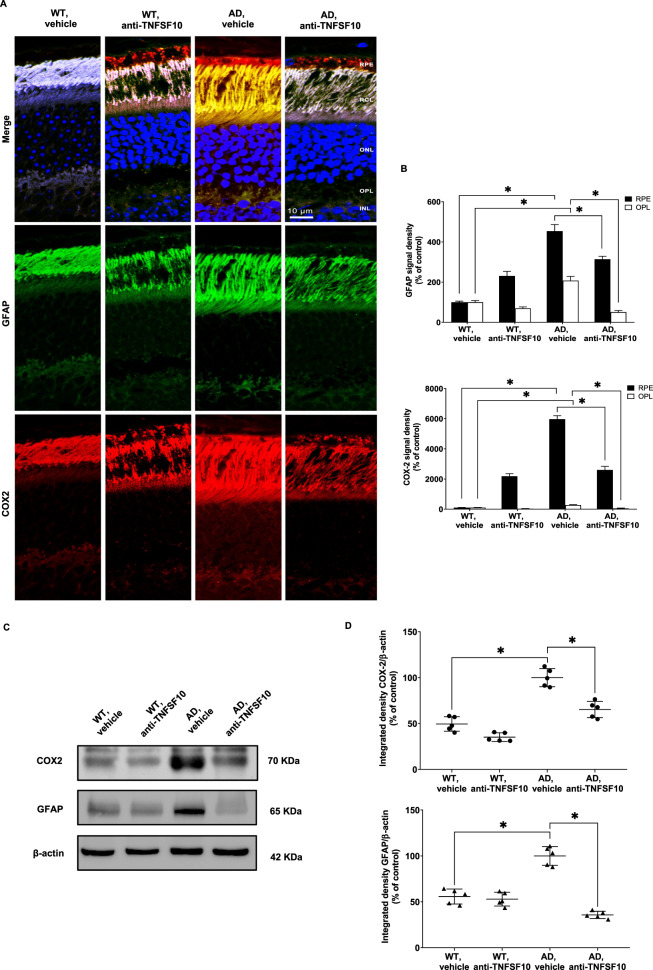
Anti-TNFSF10 treatment inhibited astrogliosis in the outer-plexiform and in the RPE layers of the 3xTg-AD mouse retina. **A** Immunohistochemical staining for GFAP, COX2 in the retina of WT and 3xTg-AD mice treated with anti-TNFSF10 or vehicle. Original magnification, x63. Scale bar = 10 µm. **B** Densitometric analysis of the GFAP and COX2 immunofluorescence signal in the RPE and OPL retinal layers. One-way ANOVA and post-hoc Tukey’s multiple comparisons test were used for statistical analysis. * *p* < 0.05. *N* = 5 animals; 5 independent retinal samples per group. For each retinal section, 14 optical fields were analyzed. **C** Western blot images for GFAP, COX-2 protein expression in the retina of mice following chronic treatment with an anti-TNFSF10 monoclonal antibody or vehicle. **D** Densitometric analysis of western blots. Data are expressed as mean ± standard deviation. One-way ANOVA and post-hoc Tukey’s multiple comparisons test were used to determine statistical significance. *p < 0.05. *N* = 5 animals; 5 independent retinal samples, 2 pooled retinas per sample in each group.

Moreover, increased glial fibrillary protein (GFAP) and COX2 immunostaining, hallmarks of reactive gliosis, another typical feature appearing during neurodegenerative processes, was observed in retinal RPE and OPL layers of 3xTg-AD mice. Significantly, reduction of both GFAP and COX2 expression occurred in 3xTg-AD mice treated with the TNFSF10 antibody (Fig. 7A, B, Supplementary Fig. 7). These proteins colocalized in both the retinal RPE and OPL layers of 3xTg-AD mice (Supplementary Fig. 8). A similar trend of expression of GFAP and COX2 was observed in western blot analysis (Fig. 7C, D). In addition, as inflammation emerges as crucial common point in AMD and AD pathogenesis, we also evaluated the expression of other inflammatory markers such as Interleukin 6 (IL-6) and Interferon-γ (IFN-γ) in the retina of 3xTg-AD mice [35]. Robust expression of both IL-6 (Supplementary Fig. 9A, B) and IFN-γ (Supplementary Fig. 9C, D) was detectable in the retina of untreated 3xTg-AD mice, while treatment with the anti-TNFSF10 antibody resulted in blunted expression of both inflammatory markers.

### Accumulation of both Aβ deposits and phosphorylated Tau (p-Tau) in the retina of 3xTg-AD mice is attenuated by anti-TNFSF10 treatment

In consideration of the well-known contribution of both Aβ_1-42_ and p-Tau [36] in 3xTg-AD mice, we investigated their expression in the retina of these mice with and without chronic anti-TNFSF10 antibody.

**Fig. 8:**
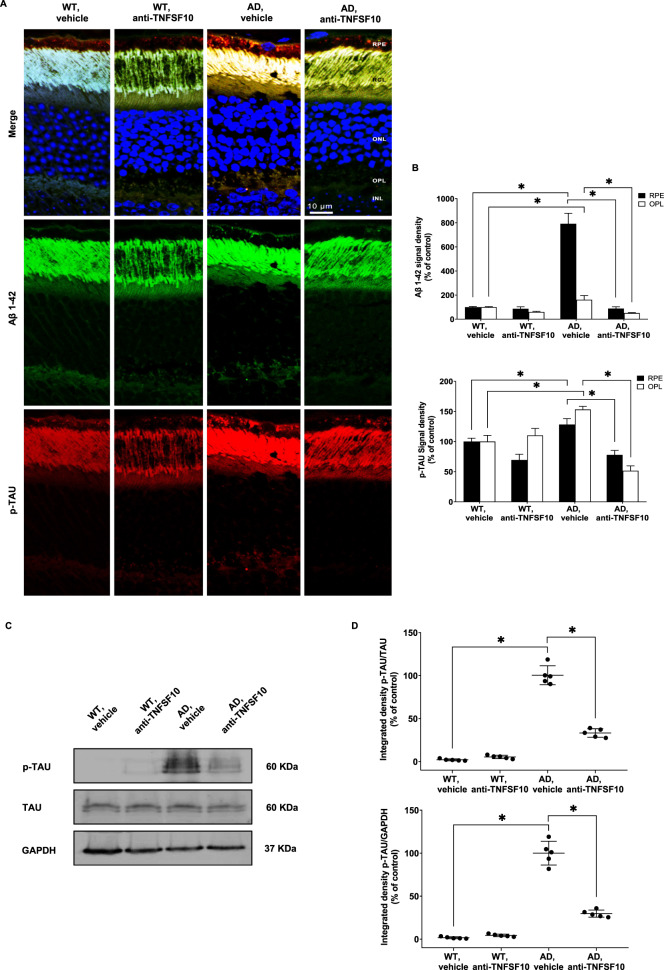
Anti-TNFSF10 treatment inhibited Aβ and p-TAU deposition in the outer-plexiform and in the RPE layers of the 3xTg-AD mouse retina. **A** Immunohistochemical staining for p-TAU in the retina of WT and 3xTg-AD mice treated with anti-TNFSF10 or vehicle. Original magnification, x63. Scale bar = 10 µm. **B** Densitometric analysis of the p-TAU immunofluorescence signal in the RPE and OPL retinal layers. One-way ANOVA and post-hoc Tukey’s multiple comparisons test were used for statistical analysis. *p < 0.05. *N* = 5 animals; 5 independent retinal samples. For each retinal section, 14 optical fields were analyzed. **C** Western blot representative images for p-TAU protein expression in the retina of mice following chronic treatment with an anti-TNFSF10 monoclonal antibody or vehicle. **D** Densitometric analysis of western blots. Data are expressed as mean ± standard deviation. One-way ANOVA and post-hoc Tukey’s multiple comparisons test were used for statistical analysis. *p < 0.05. *N* = 5 animals; 5 independent retinal samples, 2 pooled retinas per sample in each group.

Indeed, p-Tau was detected in retinas of 3xTg-AD mice and eventually colocalized with Aβ deposits in the OPL layer but especially in the RPE cell layer (Supplementary Fig. 10). A remarkable reduction of Aβ and p-Tau immunostaining was observed after treatment with a TNFSF10-neutralizing antibody (Fig. 8A, B, Supplementary Fig. 11). Western blot analysis confirmed these findings (Fig. 8C, D).

## Discussion

Circulating serum miRNAs or tissue-specific miRNAs, have been largely considered as feasible disease biomarkers in the oncology field, but also in ocular diseases [11, 37]. The expression pattern of miRNAs has been also analyzed in AD, either in pre-clinical or clinical studies [38, 39]. Several studies have investigated the role of a single miRNA (i.e., miR-181 [40, 41], miR-369 [42], miR-31 [43], miR-342 [44], miR-132/212 [45], miR-34a [46, 47], miR-155 [20], miR-146a [48]) in 3xTg-AD mice, but only a few of these studies were focused on differential expression of more than one noncoding RNA [49, 50].

Here, we evaluated the expression of a focused set of miRNAs previously validated in a rat model of AMD and in serum of AMD patients [11], in the retina of 3xTg-AD mice at different ages, instead of using high-throughput analysis. Considering that, many other retinal miRNAs could be dysregulated in this strain but also that high-throughput analyses are quite expensive and need a mandatory validation step (qPCR) [51], our focused strategy (i.e., literature search and bioinformatic validation) was aimed to increase the success rate of miRNAs and gene target analysis.

Indeed, results obtained hereby can be inferred for mechanistic and pharmacological studies in age-related ocular degenerative diseases, such as glaucoma and AMD, that share common pathogenetic mechanisms with AD [16].

We found that five miRNAs were dysregulated in the retina of 3xTg-AD mice (miR-155-5p, miR-126-3p, miR-34a-5p, miR-27a-3p, miR-23a-3p). According to previous data [46, 47], we found significant up-regulation of miR-34a only in the retinas of 3-month-old 3xTg-AD mice. With regard to miR-155, Guedes et al. in 2014 showed that this miRNA was up-regulated in the brain of 3xTg-AD mice, and it was tightly linked to astrocyte and microglia activation [20]. Consistent with this evidence, we found an age-dependent retinal up-regulation of miR-155 in 3xTg-AD mice and the highest node degree distribution with susceptibility genes in the predicted miRNA-gene network, confirming that miR-155 plays a crucial role in the regulation of several pathways of AD.

Thus, we investigated the role of the dysregulated set of miRNAs through bioinformatic approaches, unraveling a tight link with the TNFSF10 signaling pathway. Furthermore, validated interactions (Tarbase algorithm) [30] were found between miR-155 and TNFSF10 death receptor TNFRSF10B mRNA, along with SOCS-1, which is a protein involved in a negative feedback loop necessary to control the proinflammatory cytokines release [20]. Now, it is well known that cytokine signaling is overactivated in AD [17]. Low expression of SOCS-1 observed in AD, depending upon mir-155 overexpression, was associated with the sustained inflammatory process that characterizes the disease [20].

Moreover, the miR-155 upregulation in 3xTg-AD mice would represent a mechanism aimed to modulate the TNFSF10 system, in response to activation of other detrimental biological pathways. Consistently, we found that retinal miR-155 expression was significantly down-regulated in anti-TNFSF10-treated 3xTg-AD mice. These results are in line with previous studies, showing the tight relationship between the TNFSF10 pathway and miR-155 [52]. Furthermore, we found that SOCS-1 was significantly down-regulated in the retina of 3xTg-AD mice, whereas it was stabilized at basal level in animals treated with anti-TNFSF10. Overall, our results point out to relevant consequences of TNFSF10 immunoneutralization in counteracting the inflammatory/immune-response sustained by miR-155 upregulation and consequent SOCS-1 downregulation in the AD retina.

The anti-TNFSF10 treatment restored a normal morphology of retinal GCL and NFL. These results are aligned with previous studies showing changes in retinal morphology of AD patients [53] and other types of retinal degeneration [54]. In this scenario, our data are consistent with data demonstrating that immunoneutralization of TNFSF10 is correlated with neuroprotection [27, 55].

The expression of TNFSF10 and its TNFRSF10B receptor was significantly higher in the retina of untreated 3xTg-AD mice, while anti-TNFSF10 treatment resulted in significantly decreased expression of both proteins. This appears in line with other data, showing an increase of TNFSF10 and its death receptor in different neurodegenerative processes, occurring, for example, after spinal cord injury [55], and in the post-ischemic stroke [56].

As the TNFSF10 system has an orchestrating role in immune/inflammatory response during neuroinflammatory processes related to neurodegeneration [25, 28], we found a constitutively increased expression of the microglia marker Iba-1, as well as of the microglia-released cytokine TNF-α in the retina of 3xTg-AD mice. Both proteins colocalized in the RPE and the OPL layers and their colocalization was attenuated following anti-TNFSF10 treatment. This is in line with the decreased proinflammatory microglia activity shown in the brain of 3xTg-AD mice after anti-TNFSF10 treatment [28], indicating that the immunomodulating effect of TNFSF10 is extended to different areas of the central nervous system.

When an inflammatory response is triggered and sustained by arrays of proinflammatory cytokines [17], a counterbalancing anti-inflammatory response is promptly set into motion through the release of inhibitory molecules with the aim to restrain the overshoot of the inflammatory response and consequent tissue damage [25]. In a similar fashion, our results showed that the levels of the anti-inflammatory cytokine IL-10 substantially increase after the anti-TNFSF10 treatment. In this line, decreased IL-10 expression has been founded in neuroinflammatory conditions during neurodegenerative processes caused by trauma [55] or stroke [56], encompassed in its pleiotropic anti-inflammatory role in peripheral inflammatory diseases, such as rheumatoid arthritis [57], and inflammatory bowel disease [58].

Thus, it is plausible to hypothesize that the prominence of neuroinflammatory features in the retina of 3xTg-AD mice is the result of unbalanced occurrences, where the proinflammatory component gains an advantage over the anti-inflammatory one.

Consistent with the above reports, the anti-TNFSF10 treatment resulted in an increased expression of IL-10 associated with increased colocalization within Iba-1-positive cells.

A relevant contribution to neuroinflammation is given by gliosis, which corresponds to activation of repair processes associated with brain inflammation [59]. Gliosis-related overexpression of inflammatory molecules is a typical feature shared by neurodegenerative processes [60]. Gliosis implies an increased expression of its specific marker, GFAP, in the brain [61]. The anti-gliosis effect of the anti-TNFSF10 treatment observed in our experiments demonstrated that the anti-inflammatory effects of the treatment also encompassed a weaker glial response, likely responsible for the rescue of retinal cells, paralleled by the positive effects occurring in the damaged brain areas [27]. Consistently to the decreased number of activated glial cells in the retina of anti-TNFSF10 treated 3xTg-AD mice, we also observed a decreased expression of the inflammatory marker COX2, highly induced in glial cells during neurodegeneration [62].

Moreover, we observed an upregulation of both IL-6 and IFN-γ in retinal lysates from untreated 3xTg-AD mice, as expression of Aβ-induced gliosis. Both IL-6 and IFN-γ expression was significantly attenuated in 3xTg-AD mice following the anti-TNFSF10 treatment. Considering that IL-6 and IFN-γ play a significant role in the pathogenesis of AMD, and that both AD and AMD share a number of striking similarities [35], such results corroborate our hypothesis that the anti-TNFSF10 antibody treatment represents a valuable strategy for the management of sight-threatening retinal degenerative diseases.

Given the tight correlation between neuroinflammatory processes in AD and the accumulation of Aβ, as well as the presence of neurofibrillary tangles [63], we observed that the remarkable amount of retinal Aβ and p-Tau proteins in the retina of 3xTg-AD mice was significantly reduced after anti-TNFSF10 treatment. This is in line with other studies, showing that the functional outcome improvement is related to the amount of Aβ and p-Tau in the brain of 3xTg-AD [27], and that the curtailment of both the central and the peripheral immune response is followed by improvement of brain tissue parameters, along with decreased inflammatory markers and reduced amounts of anomalous proteins in discrete brain areas [28]. These findings are consistent with the hypothesis that TNFSF10 is a driver of the inflammatory/immune response in different conditions of neuronal damage [23, 27, 28, 55, 56]. Immunohistochemical analysis highlighted that the neuroinflammatory hallmarks were expressed in the OPL and in the RPE layer, while retinal histochemical analyses evidenced that anti-TNFSF10 treatment preserved other retinal layers of AD mice, such as GCL and NFL. Indeed, RPE and OPL layers are involved in AMD, and, specifically, RPE and OPL layers were thinner in subjects with early AMD and neurodegeneration [64]. Therefore, it is plausible to hypothesize that the anti-TNFSF10 treatment could exert retrograde neuroprotection and anti-inflammatory action from outer (RPE and OPL) layers to the inner retina (retinal ganglion cells), probably preventing trans-neuronal degeneration [65], and photoreceptor degeneration induced by amyloid aggregation [1, 66].

In conclusion, we demonstrated that five miRNAs were constitutively dysregulated in the retina of 3xTg-AD mice, showing an age-related expression pattern. Furthermore, we observed that miR-155 expression was significantly modulated by the anti-TNFSF10 treatment, finally resulting in reduced inflammation and neuroprotective effects on the retina of 3xTg-AD mice.

We also showed that the Aβ eye-related pathology observed in the 3xTg-AD mouse model is sustained, to a large extent, by the proapoptotic cytokine TNFSF10, in redundancy with an array of inflammatory molecules. Systemic treatment with a TNFSF10 neutralizing antibody implies a dramatic improvement in either tissue or inflammatory parameters in competent retinal cells.

Finally, our results show that neutralization of TNFSF10 brings about significant amelioration of the Aβ-related eye pathology, suggesting potential therapeutic target for AD-related and other degenerative retinal disorders. Altogether, our findings suggest that TNFSF10 could be a useful tool for immunopharmacological management of age-related ocular diseases.

## Materials and methods

### Animals

Experiments were performed in 3xTg-AD mice harboring three human mutated genes (B6129-Psen1^tm1Mpm^Tg (APPSwe, tauP30L)1Lfa/J) and age-matched wild type (WT) mice (B6129SF2/J), purchased from Jackson Laboratories (Bar Habor, ME, USA).

The 3xTg-AD mice, overexpressing mutant amyloid precursor protein (APP (APPSwe)), presenilin 1 (PSEN1 (PS1M146V)), and tau (tauP301L), were originally generated by co-injecting two independent transgene constructs encoding human APPSwe and tauP301L (4 R/0 N) (controlled by murine Thy1.2 regulatory elements) into single-cell embryos harvested from mutant homozygous PS1M146V knock-in mice, which were reimplanted into foster mothers. Wild-type mice of mixed genetic background 129/C57BL6 were used as controls. The original 3xTg-AD mice strain was generated and described by Oddo et al. [36].

Wild-type mice of mixed genetic background 129/C57BL6 were used as controls.

All animals were housed under controlled light (12 h light/night cycle), in temperature- and humidity-controlled rooms, with access to food and water ad libitum. All experiments using animals were approved by the Italian Ministry of Health and conducted in accordance to the European Community directive guidelines for the use of animals in laboratory (2010/63/EU) and the Italian law (D.Lgs. 26/2014). All procedures minimized the number of animals used and their suffering.

### Experimental groups, drug administration, and sample collection

For a first validation experiment, a panel of miRNAs was analyzed in 3xTg-AD at different time-points resembling the evolution of an AD-like pathology (3, 9, and 15 months of age) and in age-matched wild-type mice, 6 mice per group. For this experiment, two retinas, from different animals of the same group were pooled.

For drug administration study, twenty 3xTg-AD and twenty wild-type mice were enrolled at 3 months of age and four study groups were used: (1) ten wild-type mice plus vehicle (Purified Rat IgG2ακ Isotype Control; BD Biosciences, San Jose, CA, USA); ten wild-type mice plus TNFSF10-neutralizing antibody (Purified Rat Anti-Mouse CD253; BD Biosciences); (iii) ten 3xTg-AD mice plus vehicle; and (iv) ten 3xTg-AD mice plus TNFSF10-neutralizing antibody. Animals (*n* = 10 per each experimental group) were treated with TNFSF10-neutralizing antibody (concentration: 0.05 mg/ml; 200 μl/ mouse; i.p.) or vehicle (concentration: 0.05 mg/ml; 200 μl/ mouse; i.p.) twice a month and sacrificed at 15 months of age, 2-weeks after the last injection.

Given 10 mice per experimental group, 20 eyes per experimental group were isolated. Specifically for western blot analysis, 10 retinas were randomly collected from 5 different mice of the same experimental group, 2 retinas per group were pooled in a vial, then given a total of *N* = 5 independent retinal samples (biological replicates) per group. Five eye globes from 5 mice per group were used for qPCR analysis, carried out for miRNA expression analysis on anti-TNFSF10 treated and untreated mice. The contralateral remaining 5 eye globes from different 5 animals per group were fixed, then retinas were isolated to carry out hematoxylin and eosin (H&E) and immunofluorescence staining experiments.

### microRNA extraction, cDNA synthesis, and qPCR

The retina from the ocular globe was isolated and placed in RNAlater solution (Ambion Biosystems, Austin, TX, USA), stored at 4 °C overnight then transferred to −80 °C. The extraction of total RNA from mice retina samples was carried out with TRIzol Reagent (Invitrogen, Life Technologies, Carlsbad, CA), according to the manufacturer’s protocol. The A260/A280 ratio of the optical density of RNA samples (measured with Multimode Reader Flash di Varioskan™) was within 1.95–2.01. cDNA was synthesized from 10 ng of RNA with TaqMan® Advanced miRNA cDNA Synthesis Kit (ThermoFisher Scientific, Cat. No. A28007). According to the manufacturer’s instructions, the poly(A) tailing reaction has been performed, followed by the adaptor ligation reaction and by the reverse transcription (RT) reaction. Subsequently, the miR-Amp reaction was carried out to obtain the undiluted miR-Amp reaction product. The miR-Amp reaction product was diluted 1:10, and the amplification was carried out by using Taqman® Advanced MicroRNA Assays (ThermoFisher Scientific) and Taqman® Fast Advanced Master Mix (ThermoFisher Scientific, Cat. No 4444557). The miR-155-5p (mmu480953_mir), miR-126a-3p (mmu482681_mir), miR-23a-3p (mmu478532_mir), miR-34a-5p (mmu481304_mir), miR-9-5p (mmu481285_mir) and miR-27a-3p (mmu478384_mir), miR-146a-5p (mmu478399_mir) has been analyzed. The miR-16-5p (mmu482960_mir) has been used for the normalization. Real-time PCR was carried out on a 7900 HT Fast Real Time PCR System (Applied Biosystems, Monza, Italy). MicroRNA expression was quantified as -ΔCt, where Ct is the threshold cycle, and -ΔCt is the negative of Ct target miRNA minus Ct miR-16.

### Bioinformatics analysis

An integrated bioinformatic approach was carried out to predict the biological effect of the differential expression of a specific set of miRNAs, in the retina of 3xTg-AD mice compared to control wild-type mice. Specifically, the input of miRNet analysis [67] were the miRNAs significantly differentially expressed in the retina of 3xTg-AD mice, compared to the retina of age-matched WT mice (miR-155-5p, miR-126-3p, miR-34a-5p, miR-23a-3p, miR-27a-3p). Since human and murine miRNAs share high sequence homology and identity, we selected in miRNet analysis the “human” option as setting for “organism”, to characterize our analysis with a translational approach. The miRNA-target genes network was built applying the Tarbase v.8, an algorithm for the prediction of experimentally validated miRNA-mRNA target gene interactions [30]. The miRNet analysis also provided the prediction of diseases, characterized by dysregulation of the input set of miRNAs. The output of miRNet, a miRNA-target genes network, was analyzed as an undirected graph with Cytoscape, through analysis degree metrics, because of its large dimensions (more than 2000 nodes, more than 17000 undirected node-node interactions). Network analysis, i.e. centrality metrics, has been carried out accordingly to principles or network stability parameters, as previously reported [68]. Within the miRNet analysis, we then carried out an enriched analysis of mRNA-target genes network, through the “function explorer” module, setting the hypergeometric test as algorithm. Other specific analyses were carried out with DIANA tools (http://diana.imis.athena-innovation.gr/DianaTools/index.php), such as Kegg pathways enrichment [69] (https://www.genome.jp/kegg/pathway.html).

### Tissue homogenization and protein extraction

The retina samples of 3xTg-AD and age-matched wild-type mice were dissected in ice-cold Hank’s balanced salt solution (HBSS: 137 mM NaCl, 5.4 mM KCl, 0.45 mM KH2PO4, 0.34 mM Na2HPO4, 4 mM, NaHCO3, 5 mM glucose; pH 7.4), the two retinas per group were pooled and then frozen in liquid nitrogen and stored at −80 °C, until use. For protein extraction, retinal tissues were lysed in a lysis buffer containing 150 mM NaCl, 50 mM Tris–HCl (pH 7.5), 5 mM EDTA, 1 mM Na3VO4, 30 mM sodium pyrophosphate, 50 mM NaF, 1 mM acid phenyl-methyl-sulphonyl-fluoride, 5 μg/ml aprotinin, 2 μg/ml leupeptin, 1 μg/ml pepstatin, 10% glycerol, and 0.2% TritonTM X-100 and sonicated with 3 pulses of 2 s each. The homogenates were then centrifuged at 14,000 rpm for 10 min at 4 °C and the supernatant was collected. The protein concentration of the supernatant was determined by the Bradford method [70].

### Western blot analysis

Equal amounts of protein (40 µg) were resolved by 8–12% SDS-PAGE gels and transferred onto Hybond ECL nitrocellulose membranes (GE Healthcare, Little Chalfont, UK). Membranes were blocked for 1 h at RT with 5% nonfat dry milk or 5% BSA in phosphate-buffered saline plus 0.1% Tween 20 (PBS-T). For primary antibody reactions, a rabbit anti-SOCS1 (Cell Signaling Technology Inc., Danvers, MA, USA), or a rabbit anti-TNFRSF10B (Abcam, Cambridge, UK), or a rabbit anti-TNFSF10 (Abcam), or a mouse anti-Iba1 (Abcam), or a rabbit anti-TNF-α antibody (Novus Biologicals), or a rabbit anti-IL10 antibody (Abbiotec, San Diego, CA, USA), or a mouse GFAP (Cell Signaling Technology Inc.), or a mouse anti-COX-2 (Santa Cruz Biotechnology Inc., Santa Cruz, CA, USA), or a mouse anti-p-Tau antibody (Santa Cruz Biotechnology Inc.), or a rabbit anti-Tau antibody (Santa Cruz Biotechnology Inc.), or a mouse IFN-γ (Santa Cruz Biotechnology Inc.), or anti-rabbit IL-6 (Cell Signaling Technology Inc.) were added to membranes and stayed overnight at 4 °C on an orbital shaker. Then, the membranes were washed with PBS-T and were probed with the appropriate horseradish peroxidase-conjugated anti-rabbit or anti-mouse IgG antibody (Amersham Life Science, Buckinghamshire, UK) for 1 h at RT. Beta-Tubulin or β-actin (Santa Cruz Biotechnology Inc.) or GAPDH (Cell Signaling Technology Inc.) were used as control to validate the amount of protein loaded in the gels. After washing with PBS-T, protein bands were visualized by enhanced chemiluminescence (Thermo Fisher Scientific) and scanned with the iBright FL1500 Imaging System (Thermo Fisher Scientific). Densitometric analysis of band intensity was done on immunoblots by using IMAGE J software (https://imagej.nih.gov/ij/). Full details of the antibodies used are reported in Supplementary Table 2.

### Hematoxylin and eosin (H&E) staining

Retinal tissue samples were fixed in 10% neutral-buffered formalin (Bio-Optica) for 24 h. After overnight washing, tissue samples were dehydrated in graded ethanol and paraffin-embedded. Sections of 4–6 μm in thickness were cut and mounted on silanized glass slides and air-dried. To remove the paraffin, slides were immersed in xylene two times, for 3 min each; rehydrated with graded ethanol, 100%, 95%, 80%, 70%, and 50%, for 3 min each; and transferred to tap water.

After that, tissues were stained with (H&E) and morphological examination of the samples was performed using an Axioplan Zeiss light microscope (Germany).

### Immunofluorescence

After collection, eye globes were fixed in 4% w/v paraformaldehyde in phosphate buffer saline 0.1 M pH 7.4 (PBS) for 2 h at room temperature. Retinal tissues paraffin-embedded were cut in 5 μm sections and placed on glass slides. After deparaffinization and rehydration, tissue specimens were processed as previously described [28] with a few modifications. Briefly, after antigen retrieval in sodium citrate buffer (10 mM sodium citrate, 0.05% Tween-20, pH 6.0)) by microwave for 15 min, slides were washed in PBS containing 0.25% Triton X-100 (PBST) twice for 5 min each, blocked in 1% BSA in PBST for 1 h at RT, briefly rinsed with PBST and incubated for 1 h at RT with the following primary antibodies: a goat anti-TNFRSF10B (Abcam), or a rabbit anti-TNFSF10 (Abcam), or a mouse anti-Iba1 (Abcam), or a rabbit anti-TNF-α antibody (Novus Biologicals), or a rabbit anti-IL10 antibody (Abbiotec), or a rabbit GFAP (Abcam), or a mouse COX-2 (Santa Cruz Biotechnology Inc.), or a mouse anti-p-Tau antibody (Santa Cruz Biotechnology Inc.). For immunopositive reactions and fluorescence detection, after washing in PBS three times for 5 min each, sections were incubated using the appropriate fluorescent-labeled secondary antibodies (Invitrogen; Thermo Fisher Scientific, Inc, MA, USA) at dark for 1 h at RT. See Supplementary Table 2 for full details of the antibodies used. Finally, for nuclear staining, slides were washed and mounted with DAPI-containing mounting solution (Fluoroshield with DAPI; Sigma-Aldrich, Milan, Italy) and secured with a coverslip. Images were observed using a laser scanning confocal microscope (Zeiss LSM 700, Germany) and ZEN2010 software was used for image acquisition and colocalization analysis. Intensity level of the fluorescent signals was evaluated using the ImageJ software (NIH, Bethesda, MD; available at http://rsb.info.nih.gov/ij/index.html). Mean data from 14 optical fields (4 × 4 µm of 5-µm-thick sections) were analyzed with one-way ANOVA followed by Tukey’s test post hoc analysis. Differences between groups were considered significant at *p < 0.05.

### Statistical evaluation

Investigators that carried out treatment and analyses were blinded to group labels. Group labels were unveiled after draft graph design and statistical analyses. Sample size was chosen considering the calculation provided by power analysis and the possibility that mice would die or be excluded within 15 months-long experimental protocol. For animals and relative samples, the exclusion criteria from experimental protocol were: sudden death, loss of weight >20%, sign of distress (eyes squinted, contraction of the skin around the nose, ears pulled back, and lethargy or non-responsiveness). Within the monitoring of animal health during the experiment, no animals or samples were excluded from the study. Specifically, given the lowest expected difference between the means of two groups and homogeneous variance within the groups, the calculated sample size was *n* = 4, for 1-β set to 0.80 and α set to 0.05 (G*power software) [71]. The number of animals and independent retinal samples (biological replicates) used was *n* = 6 for miRNAs expression analyses and *n* = 5 for the other analyses, see the “Experimental groups, and drug administration and sample collection” paragraph in the methods section. Data were analyzed to test normality distribution. Data were represented as mean±standard deviation (SD), from at least three independent samples, and three technical replicates. Data were analyzed by the one-way analysis of variance (ANOVA) test, followed by the Tukey post-hoc test for multiple comparisons. Post-hoc tests were carried out only if F had a *p* < 0.05, and no significant variance in homogeneity was found within the analyzed groups. Significance was set at a *p* < 0.05. Graph design and statistical analyses were carried out with SPSS (https://www.ibm.com/analytics/spss-statistics-software) and GraphPad Prism (https://www.graphpad.com/scientific-software/prism/).

## Supplementary information

Supplementary Table 1

Supplementary Table 2

Supplementary Figure 1

Supplementary Figure 2

Supplementary Figure 3

Supplementary Figure 4

Supplementary Figure 5

Supplementary Figure 6

Supplementary Figure 7

Supplementary Figure 8

Supplementary Figure 9

Supplementary Figure 10

Supplementary Figure 11

Supplementary figure legends

## Data Availability

The datasets used and/or analyzed during the current study are available from the corresponding author on reasonable request.
