# Supplementary material for: Targeting the miRNA-155/TNFSF10 network restrains inflammatory response in the retina in a mouse model of Alzheimer’s disease
Source: Cell Death Dis. 2021 Oct 5;12(10):905. doi: 10.1038/s41419-021-04165-x (PMC8492692; doi:10.1038/s41419-021-04165-x)
Supplement: Supplementary file 1 — Supplementary Table 1 [file 41419_2021_4165_MOESM1_ESM.doc]

**Supplementary Table 1. Enriched pathways from miRNET miRNA-gene interaction network.** Highlighted pathways include genes of the TNFSF10 signaling network.

| **Pathways** | **genes** | **p-value** |
| --- | --- | --- |
| Alcoholism | 78 | 1.5e-19 |
| Pathways in cancer | 104 | 4.08e-13 |
| Colorectal cancer | 29 | 4.1e-11 |
| Prostate cancer | 41 | 7.71e-11 |
| Pancreatic cancer | 33 | 3.55e-9 |
| Apoptosis | 37 | 4.6e-9 |
| Chronic myeloid leukemia | 34 | 4.88e-9 |
| HTLV-I infection | 67 | 7.39e-9 |
| Endometrial cancer | 23 | 1.1e-7 |
| Toxoplasmosis | 37 | 1.78e-7 |
| Non-small cell lung cancer | 25 | 2.56e-7 |
| T cell receptor signaling pathway | 38 | 2.68e-7 |
| Adherens junction | 30 | 3.94e-7 |
| Cell cycle | 44 | 5.97e-7 |
| p53 signaling pathway | 29 | 6.99e-7 |
| Glioma | 28 | 8.5e-7 |
| Neurotrophin signaling pathway | 41 | 0.00000888 |
| Small cell lung cancer | 30 | 0.0000109 |
| Huntington's disease | 15 | 0.0000128 |
| Bladder cancer | 15 | 0.0000223 |
| Melanoma | 26 | 0.0000282 |
| Acute myeloid leukemia | 23 | 0.0000298 |
| Chagas disease (American trypanosomiasis) | 31 | 0.0000426 |
| Osteoclast differentiation | 38 | 0.0000558 |
| Influenza A | 35 | 0.0000629 |
| Bacterial invasion of epithelial cells | 22 | 0.0000719 |
| Amyotrophic lateral sclerosis (ALS) | 17 | 0.000105 |
| Lysine degradation | 19 | 0.000142 |
| Legionellosis | 17 | 0.000153 |
| B cell receptor signaling pathway | 26 | 0.000191 |
| Toll-like receptor signaling pathway | 31 | 0.000263 |
| Focal adhesion | 54 | 0.000288 |
| Epstein-Barr virus infection | 29 | 0.000435 |
| Shigellosis | 18 | 0.000469 |
| Hepatitis C | 31 | 0.000479 |
| Viral myocarditis | 12 | 0.000586 |
| Renal cell carcinoma | 21 | 0.000673 |
| NOD-like receptor signaling pathway | 18 | 0.00084 |
| Jak-STAT signaling pathway | 30 | 0.000896 |
| Wnt signaling pathway | 40 | 0.000956 |
| ErbB signaling pathway | 27 | 0.00107 |
| Insulin signaling pathway | 38 | 0.00131 |
| Thyroid cancer | 12 | 0.00132 |
| Synaptic vesicle cycle | 9 | 0.00143 |
| Measles | 30 | 0.00153 |
| Dorso-ventral axis formation | 7 | 0.00158 |
| Arrhythmogenic right ventricular cardiomyopathy (ARVC) | 7 | 0.00158 |
| Salmonella infection | 23 | 0.0016 |
| RNA transport | 35 | 0.00195 |
| Regulation of actin cytoskeleton | 47 | 0.00196 |
| Epithelial cell signaling in Helicobacter pylori infection | 14 | 0.00226 |
| TGF-beta signaling pathway | 25 | 0.00307 |
| Herpes simplex infection | 29 | 0.00372 |
| Alzheimer's disease | 16 | 0.00627 |
| Fc epsilon RI signaling pathway | 22 | 0.00643 |
| Progesterone-mediated oocyte maturation | 23 | 0.00705 |
| VEGF signaling pathway | 22 | 0.00762 |
| MAPK signaling pathway | 61 | 0.00827 |
| Valine, leucine and isoleucine degradation | 14 | 0.0131 |
| RIG-I-like receptor signaling pathway | 15 | 0.0151 |
| mTOR signaling pathway | 14 | 0.0161 |
| Chemokine signaling pathway | 44 | 0.019 |
| Carbohydrate digestion and absorption | 7 | 0.0245 |
| Malaria | 5 | 0.0281 |
| Tuberculosis | 40 | 0.0297 |
| Fatty acid metabolism | 12 | 0.0329 |
| Rheumatoid arthritis | 7 | 0.0332 |
| Adipocytokine signaling pathway | 17 | 0.0347 |
| Fc gamma R-mediated phagocytosis | 24 | 0.0377 |
| Leukocyte transendothelial migration | 26 | 0.0429 |
| Sphingolipid metabolism | 13 | 0.043 |
| Leishmaniasis | 14 | 0.0459 |
| African trypanosomiasis | 8 | 0.0531 |
| Pyrimidine metabolism | 24 | 0.0574 |
| Melanogenesis | 24 | 0.0574 |
| Natural killer cell mediated cytotoxicity | 31 | 0.0669 |
| Tight junction | 27 | 0.0689 |
| PPAR signaling pathway | 16 | 0.0737 |
| Protein processing in endoplasmic reticulum | 29 | 0.0739 |
| Endocytosis | 23 | 0.092 |
| Transcriptional misregulation in cancer | 6 | 0.0942 |
| Notch signaling pathway | 12 | 0.0984 |
| Pertussis | 13 | 0.1 |
| Aldosterone-regulated sodium reabsorption | 9 | 0.118 |
| Hypertrophic cardiomyopathy (HCM) | 7 | 0.126 |
| Regulation of autophagy | 3 | 0.146 |
| Axon guidance | 25 | 0.154 |
| Hedgehog signaling pathway | 13 | 0.157 |
| SNARE interactions in vesicular transport | 7 | 0.171 |
| Glycerolipid metabolism | 12 | 0.174 |
| Basal cell carcinoma | 11 | 0.175 |
| Parkinson's disease | 5 | 0.187 |
| Synthesis and degradation of ketone bodies | 3 | 0.192 |
| Type II diabetes mellitus | 11 | 0.194 |
| Glyoxylate and dicarboxylate metabolism | 5 | 0.22 |
| One carbon pool by folate | 5 | 0.22 |
| Intestinal immune network for IgA production | 7 | 0.223 |
| Autoimmune thyroid disease | 3 | 0.242 |
| Cytokine-cytokine receptor interaction | 48 | 0.254 |
| Cytosolic DNA-sensing pathway | 5 | 0.255 |
