# Supplementary material for: Targeting the miRNA-155/TNFSF10 network restrains inflammatory response in the retina in a mouse model of Alzheimer’s disease
Source: Cell Death Dis. 2021 Oct 5;12(10):905. doi: 10.1038/s41419-021-04165-x (PMC8492692; doi:10.1038/s41419-021-04165-x)
Supplement: Supplementary file 2 — Supplementary Table 2 [file 41419_2021_4165_MOESM2_ESM.docx]

**Supplementary Table 2. List of antibodies used**

| **Antibodies** | **Diluition for WB** | **Dìluition**  **for IHF** | **Company (Catalog#)** |
| --- | --- | --- | --- |
| Rabbit polyclonal Anti-SOCS-1 | 1:500 | - | Cell Signaling Technology (#3950) |
| Rabbit polyclonal Anti-TNFRSF10B | 1:500 | - | Abcam (ab8416) |
| Goat polyclonal Anti-TNFRSF10B | - | 1:200 | Alexis Biochemicals (ALX-210-743) |
| Rabbit polyclonal Anti-TNFSF10 | 1:200 | 1:200 | Abcam (Ab2435) |
| Rabbit polyclonal Anti-TNF-α | 1:1000 | 1:200 | Novus Biologicals (Nb600587) |
| Rabbit poyclonal Anti-IL-10 | 1:500 | 1:250 | Abbiotec (250713) |
| Mouse monoclonal Anti-Iba-1 | 1:500 | 1:200 | Abcam (ab15690) |
| Mouse monoclonal Anti-COX-2 | 1:500 | 1:200 | Santa Cruz Biotechnology (sc-19999) |
| Rabbit polyclonal Anti-GFAP | - | 1:500 | Abcam (ab7260) |
| Mouse monoclonal Anti-GFAP | 1:500 | - | Cell Signaling Technology (#3670) |
| Mouse monoclonal Anti-IFN-γ | 1:500 | - | Santa Cruz Biotechnology (sc-59992) |
| Rabbit monoclonal Anti-IL-6 | 1:500 | - | Cell Signaling Technology (#12912S) |
| Mouse monoclonal Anti-β-actin | 1:1000 | - | Santa Cruz Biotechnology (sc-47778) |
| Mouse monoclonal Anti-β-tubulin | 1:1000 | - | Santa Cruz Biotechnology (sc-5274) |
| Rabbit monoclonal Anti-GAPDH | 1:1000 | - | Cell Signaling Biotechnology (#2118S) |
| Rabbit polyclonal Anti-Tau | 1:500 | - | Santa Cruz Biotechnology (sc-5587) |
| Mouse monoclonal Anti-p-Tau | 1:250 | 1:200 | Santa Cruz Biotechnology (sc-32275) |
| Rabbit polyclonal Anti-β-amyloid 1-42 | - | 1:500 | Merck Millipore (AB5078P) |
| Rabbit IgG HRP Linked Whole Ab | 1:5000 | - | GE Healthcare (GENA934) |
| Mouse IgG HRP Linked Whole Ab | 1:5000 | - | GE Healthcare (GENA931) |
| Alexa Fluor 488 goat anti-rabbit IgG | - | 1:500 | Life Technologies (A11008) |
| Alexa Fluor 488 donkey anti-goat IgG | - | 1:500 | Life Technologies (A32814) |
| Alexa Fluor 546 donkey anti-rabbit IgG | - | 1:500 | Life Technologies (A10040) |
| Alexa Fluor 488 goat anti-mouse IgG | - | 1:500 | Life Technologies (A11029) |
| Alexa Fluor 546 donkey anti-mouse IgG | - | 1:500 | Life Technologies (A10036) |
