# Supplementary figures and images for: Targeting the miRNA-155/TNFSF10 network restrains inflammatory response in the retina in a mouse model of Alzheimer’s disease
Source: Cell Death Dis. 2021 Oct 5;12(10):905. doi: 10.1038/s41419-021-04165-x (PMC8492692; doi:10.1038/s41419-021-04165-x)

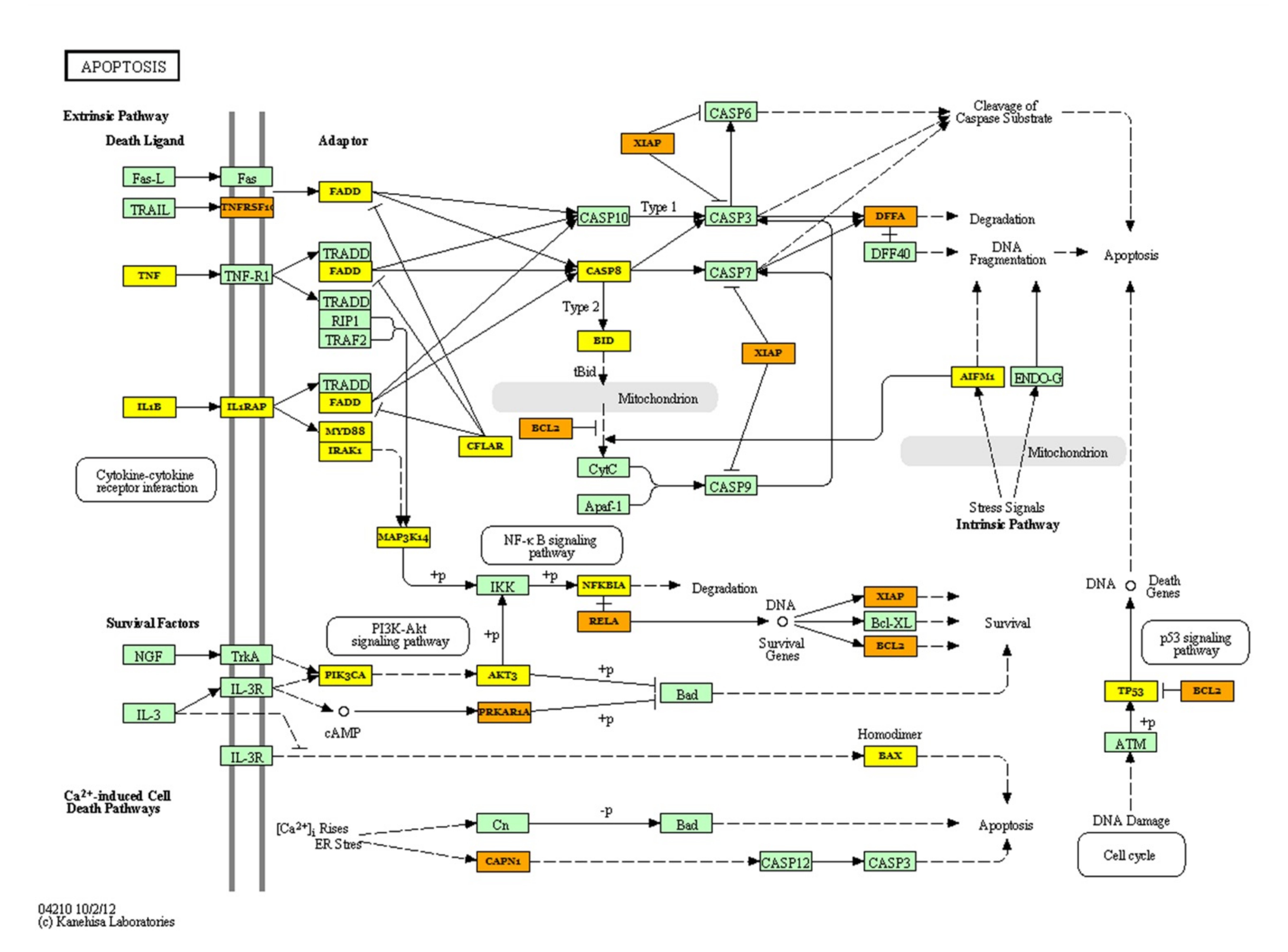

Supplement: Supplementary file 3 — Supplementary Figure 1 [file 41419_2021_4165_MOESM3_ESM.tif]

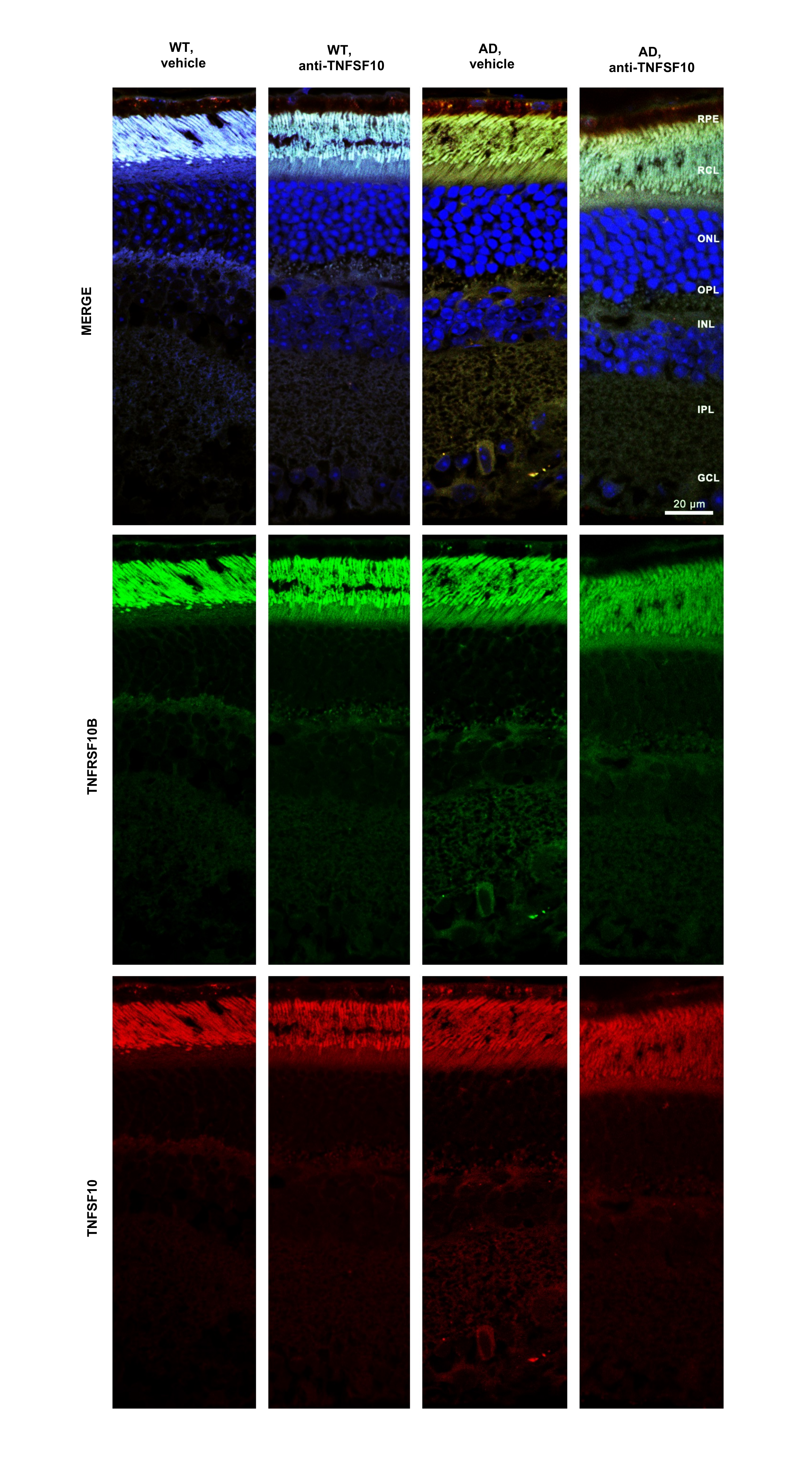

Supplement: Supplementary file 4 — Supplementary Figure 2 [file 41419_2021_4165_MOESM4_ESM.tif]

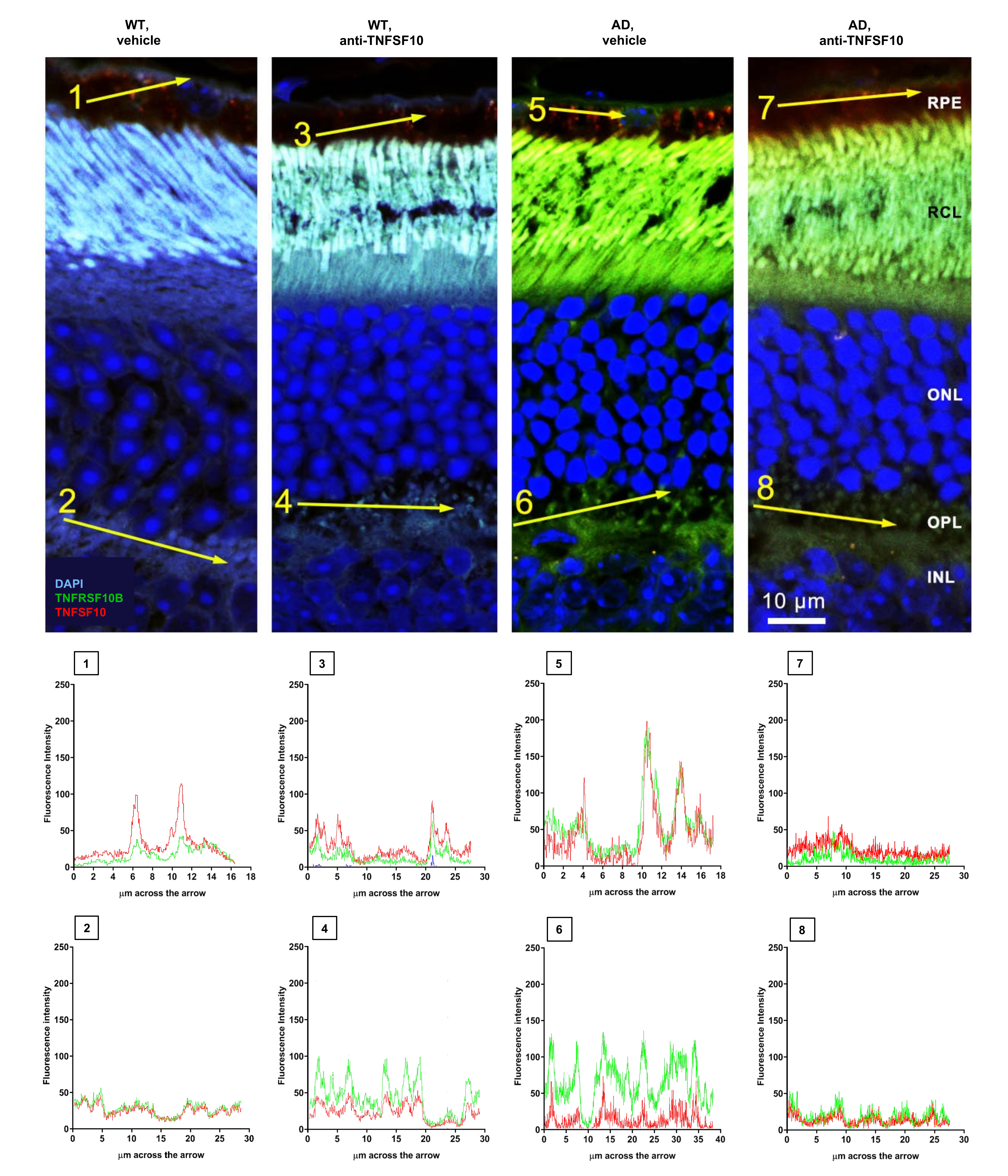

Supplement: Supplementary file 5 — Supplementary Figure 3 [file 41419_2021_4165_MOESM5_ESM.tif]

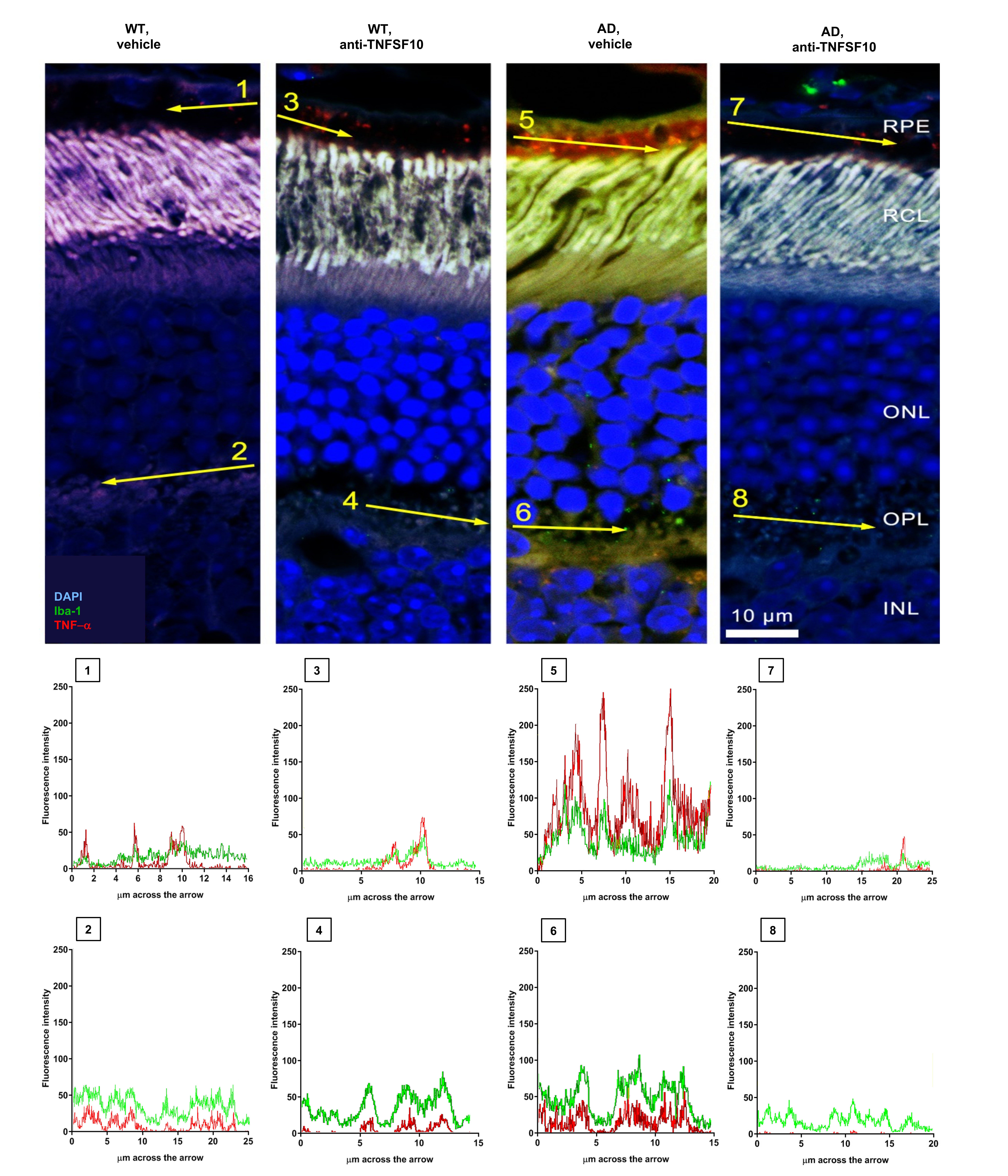

Supplement: Supplementary file 6 — Supplementary Figure 4 [file 41419_2021_4165_MOESM6_ESM.tif]

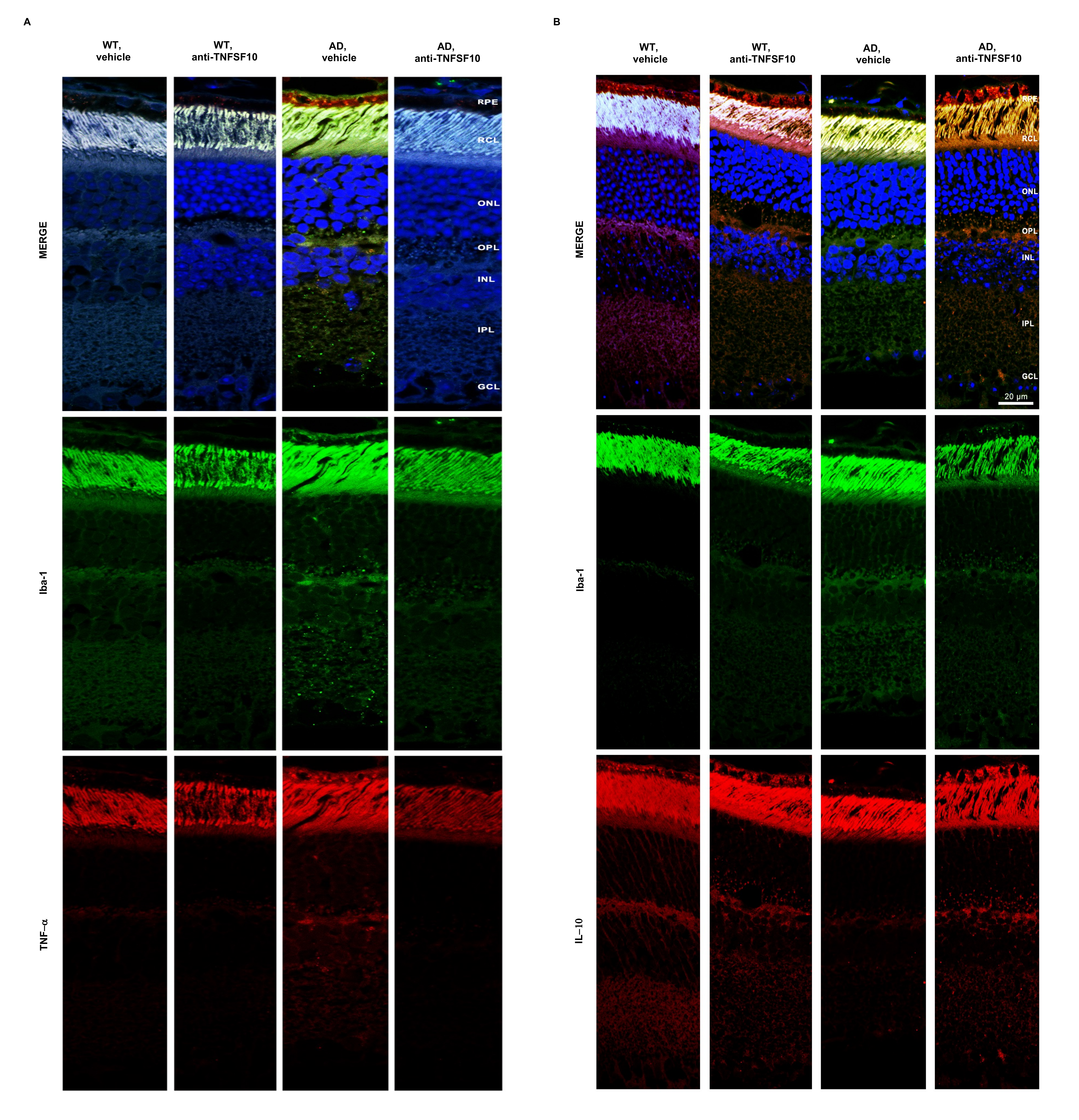

Supplement: Supplementary file 7 — Supplementary Figure 5 [file 41419_2021_4165_MOESM7_ESM.tif]

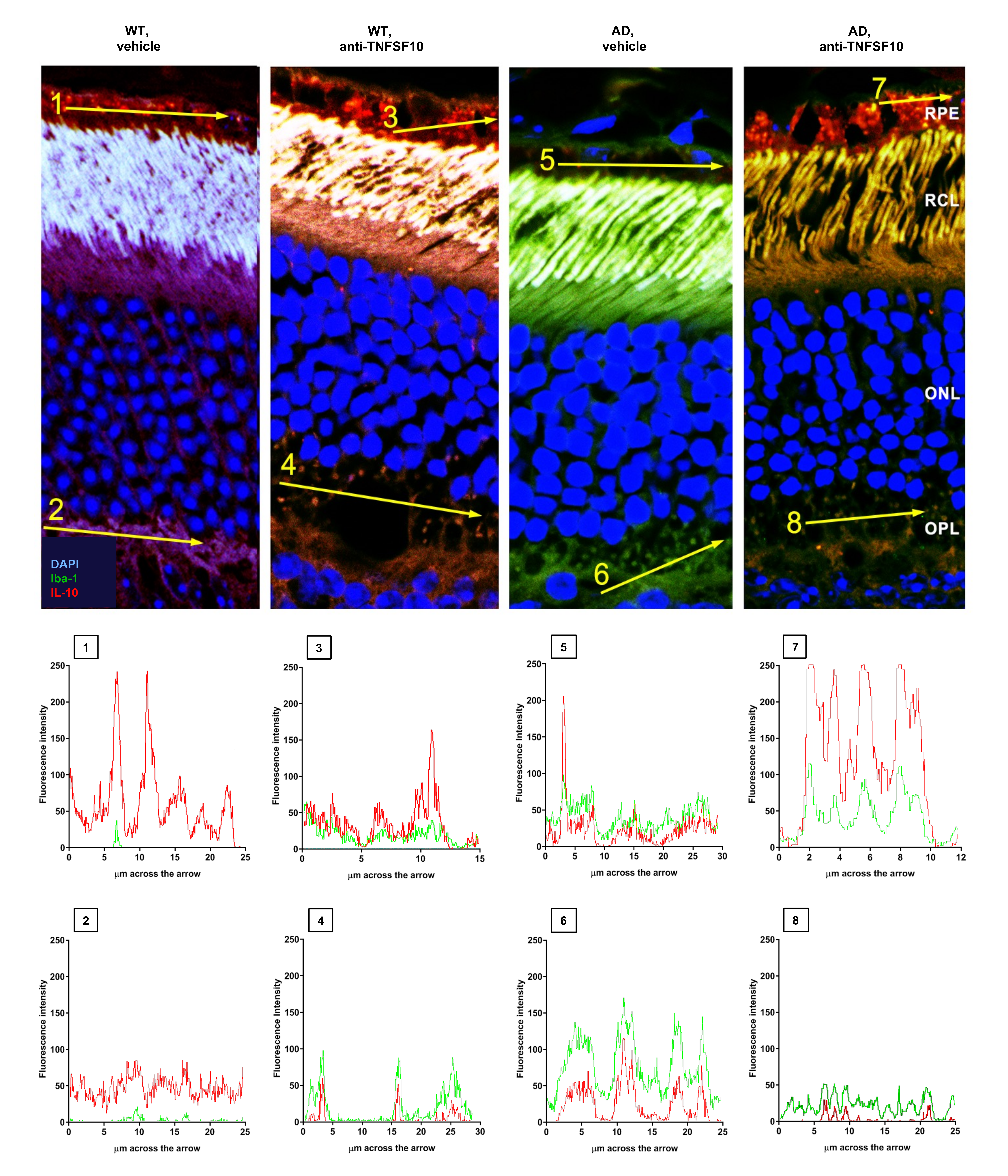

Supplement: Supplementary file 8 — Supplementary Figure 6 [file 41419_2021_4165_MOESM8_ESM.tif]

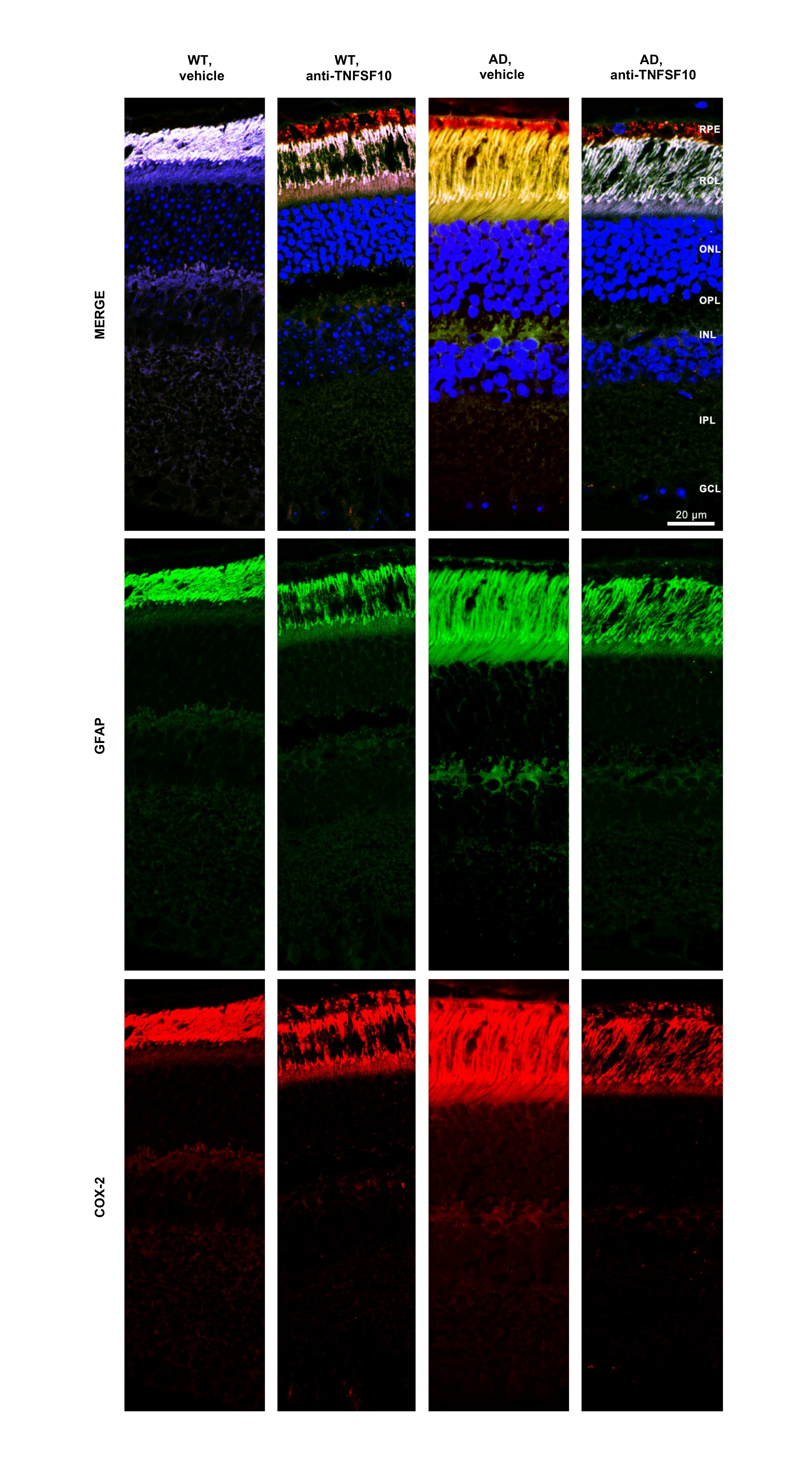

Supplement: Supplementary file 9 — Supplementary Figure 7 [file 41419_2021_4165_MOESM9_ESM.tif]

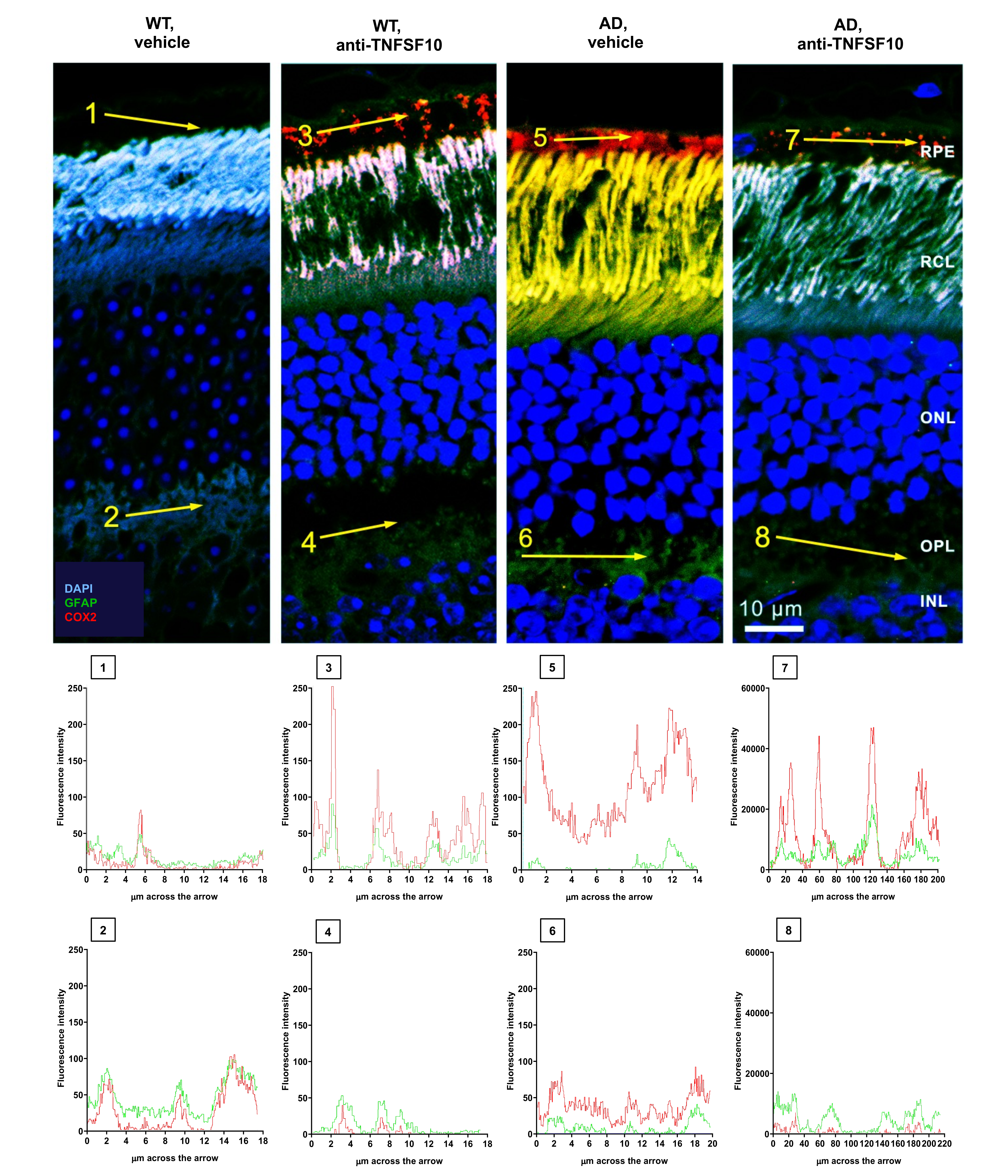

Supplement: Supplementary file 10 — Supplementary Figure 8 [file 41419_2021_4165_MOESM10_ESM.tif]

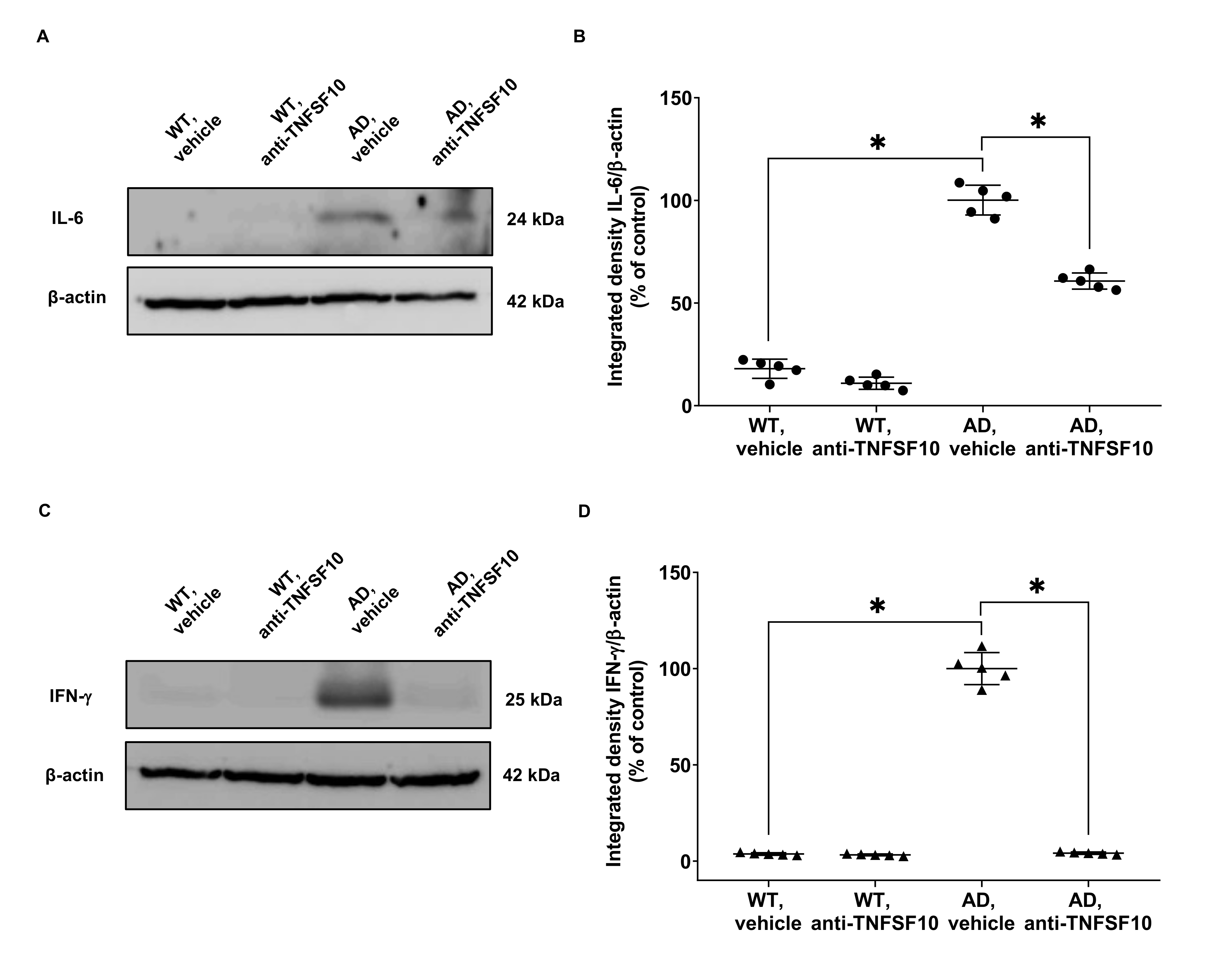

Supplement: Supplementary file 11 — Supplementary Figure 9 [file 41419_2021_4165_MOESM11_ESM.tif]

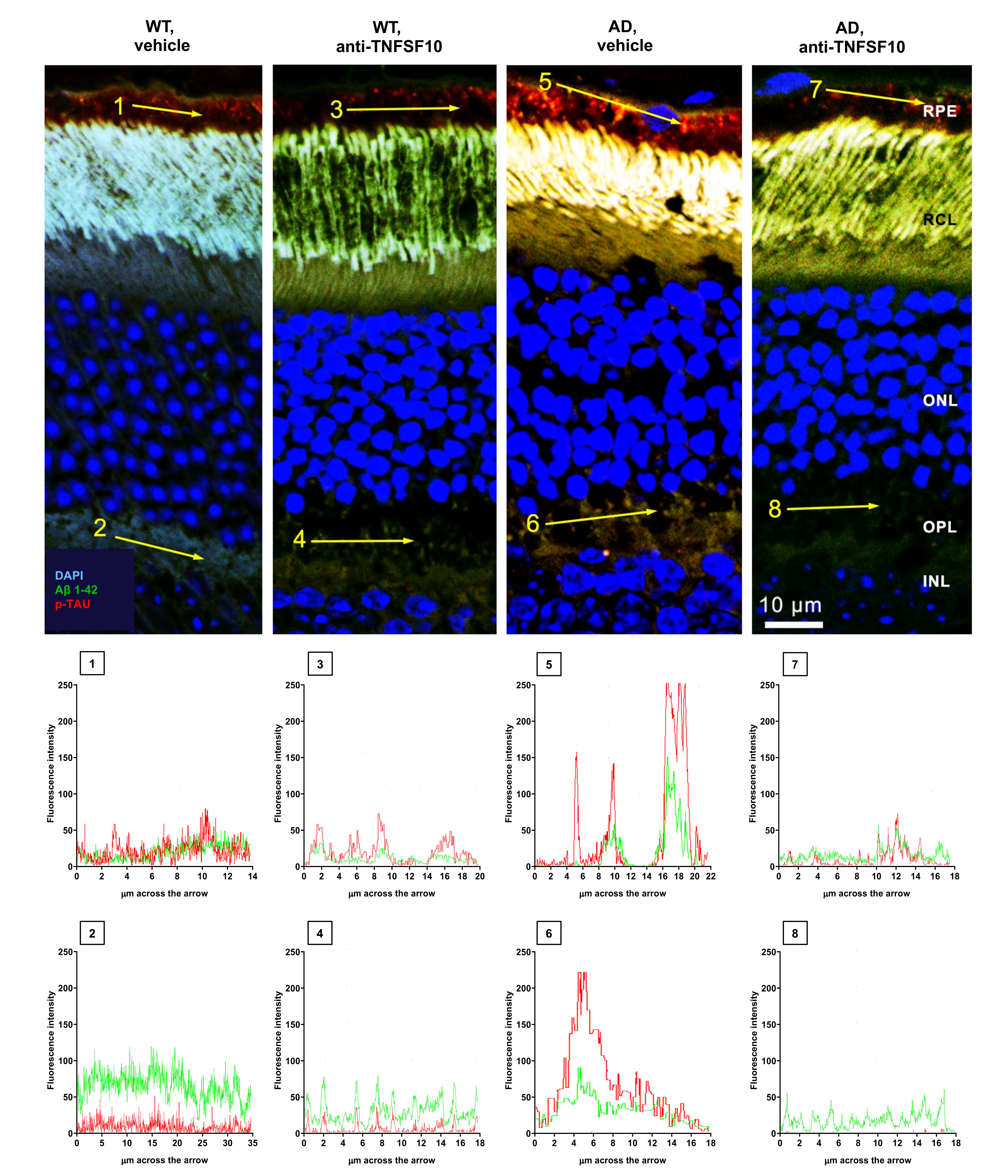

Supplement: Supplementary file 12 — Supplementary Figure 10 [file 41419_2021_4165_MOESM12_ESM.tif]

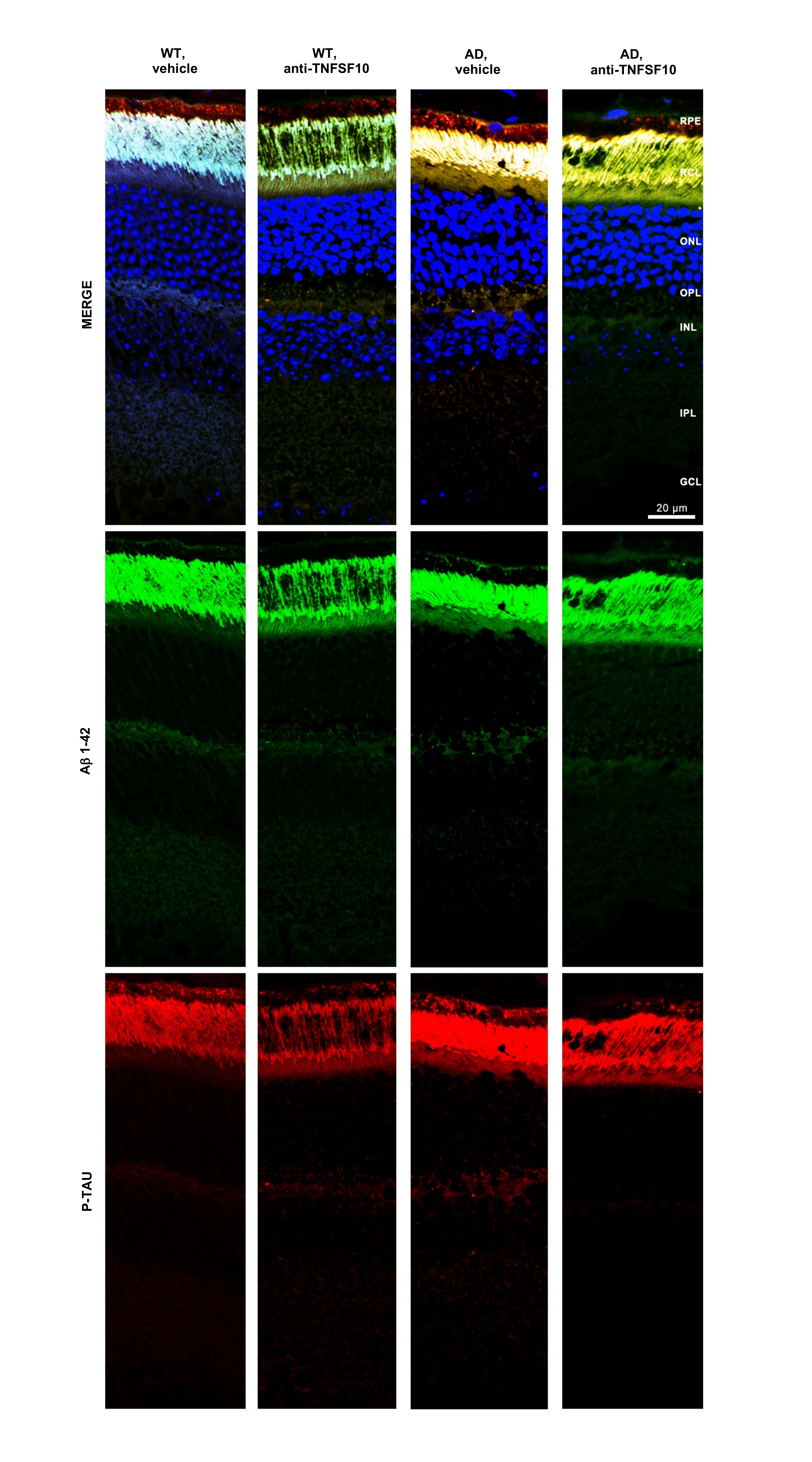

Supplement: Supplementary file 13 — Supplementary Figure 11 [file 41419_2021_4165_MOESM13_ESM.tif]
