# Supplementary material for: Targeting the miRNA-155/TNFSF10 network restrains inflammatory response in the retina in a mouse model of Alzheimer’s disease
Source: Cell Death Dis. 2021 Oct 5;12(10):905. doi: 10.1038/s41419-021-04165-x (PMC8492692; doi:10.1038/s41419-021-04165-x)
Supplement: Supplementary file 14 — Supplementary figure legends [file 41419_2021_4165_MOESM14_ESM.docx]

**Supplementary Figure Legends**

**Supplementary Figure 1. The miR-155, miR-126, miR-23a target TNFSF10 receptors TNFRSF10B (DR5) and TNFRSF10A (DR4) encompassed in the “Apoptosis” pathway.** Yellow genes are targets of one miRNA, while orange genes are targets of more than one miRNA. This picture is the output of Diana miRPath software and recalls the KEGG pathway deposited in the database (KEGG permission n°210724): https://www.genome.jp/kegg/pathway.html.

**Supplementary Figure 2. Anti-TNFSF10 treatment modulated retinal expression of TNFSF10 and its TNFRSF10B death receptor in 3xTg-AD mice.** Representative immunofluorescent staining for TNFSF10 and its receptor TNFRSF10B in the retina of WT and 3xTg-AD mice, treated with either vehicle or anti-TNFSF10 antibody. Original magnification, ×20. Scale bar = 20 µm.

**Supplementary Figure 3. Colocalization of TNFRSF10B and TNFSF10.** Upper panels show the same images presented in Fig. 5 (merged images) with the detection of TNFRSF10B (green), and TNFSF10 (red) in the control and treated retina. Bottom panels 1 to 8 show the histogram fluorescence intensity profiles across the yellow arrows for both green line (TNFRSF10B) and red line (TNFSF10) channels. The coincidence of red and green peaks indicates the colocalization of the two epitopes in that position. Original magnification x63. Scale bar = 10 µm.

**Supplementary Figure 4. Colocalization of Iba-1 and TNF-α.** Upper panels show the same images presented in Fig. 6 (merged images) with the detection of Iba-1 (green), and TNF-α (red) in the control and treated retina. Bottom panels 1 to 8 show the histogram fluorescence intensity profiles across the yellow arrows for both green line (Iba-1) and red line (TNF-α) channels. The coincidence of red and green peaks indicates the colocalization of the two epitopes in that position. Original magnification x63. Scale bar = 10 µm.

**Supplementary Figure 5. Anti-TNFSF10 treatment inhibited pro-inflammatory microglia activation in retinal layers of 3xTg-AD mouse. A)** Immunohistochemical staining for Iba-1, TNF-α in retina of WT and 3xTg-AD mice, treated with either vehicle or anti-TNFSF10 antibody. B) Immunohistochemical staining for Iba-1, IL-10 in the retina of WT and 3xTg-AD mice, treated with either vehicle or anti-TNFSF10 antibody. Original magnification, x20 for all. Scale bar = 20 µm.

**Supplementary Figure 6. Colocalization of Iba1 and IL-10.** Upper panels show the same images presented in Fig. 7 (merged images) with the detection of Iba-1 (green), and IL-10 (red) in the control and treated retina. Bottom panels 1 to 8 show the histogram fluorescence intensity profiles across the yellow arrows for both green line (Iba-1) and red line (IL-10) channels. The coincidence of red and green peaks indicates the colocalization of the two epitopes in that position. Original magnification x63. Scale bar = 10 µm.

**Supplementary Figure 7.** **Anti-TNFSF10 treatment inhibited astrogliosis in retinal layers of the 3xTG-AD mouse.** Immunohistochemical staining for GFAP, COX2 in the retina of WT and 3xTg-AD mice treated with anti-TNFSF10 or vehicle. Original magnification, x20 for all. Scale bar = 20 µm.

**Supplementary Figure 8. Colocalization of GFAP and COX2.** Upper panels show the same images presented in Fig. 8 (merged images) with the detection of GFAP (green), and COX2 (red) in the control and treated retina. Bottom panels 1 to 8 show the histogram fluorescence intensity profiles across the yellow arrows for both green line (GFAP) and red line (COX2) channels. The coincidence of red and green peaks indicates the colocalization of the two epitopes in that position. Original magnification x63. Scale bar = 10 µm.

**Supplementary Figure 9:** **Anti-TNFSF10 treatment modulated retinal expression of** **IFN-γ and IL-6 in 3xTg-AD mice.** A) Immunoblots of retinal lysates for the expression of IFN-γ and IL-6 proteins. B) Densitometric analysis of western blots. Data are expressed as mean ± standard deviation. One-way ANOVA and post-hoc Tukey’s multiple comparisons test were used to determine statistical significance. * p<0.05. N=5 animals and 5 independent retinal samples, 2 pooled retinas per sample, in each group.

**Supplementary Figure 10. Colocalization of Aβ 1−42 and p-TAU.** Upper panels show the same images presented in Fig. 9 (merged images) with the detection of Aβ 1−42 (green), and p-TAU (red) in the control and treated retina. Bottom panels 1 to 8 show the histogram fluorescence intensity profiles across the yellow arrows for both green line (Aβ 1−42) and red line (p-TAU) channels. The coincidence of red and green peaks indicates the colocalization of the two epitopes in that position. Original magnification x63. Scale bar = 10 µm.

**Supplementary Figure 11.** **Anti-TNFSF10 treatment inhibited Aβ and p-TAU deposition in retinal layers of the 3xTG-AD mouse.** Immunohistochemical staining for Aβ 1-42, p-TAU in the retina of WT and 3xTg-AD mice treated with anti-TNFSF10 or vehicle. Original magnification, x20 for all. Scale bar = 20 µm.
